# Nanocelluloses: Sources, Pretreatment, Isolations, Modification, and Its Application as the Drug Carriers

**DOI:** 10.3390/polym13132052

**Published:** 2021-06-23

**Authors:** Valentino Bervia Lunardi, Felycia Edi Soetaredjo, Jindrayani Nyoo Putro, Shella Permatasari Santoso, Maria Yuliana, Jaka Sunarso, Yi-Hsu Ju, Suryadi Ismadji

**Affiliations:** 1 Department of Chemical Engineering, Widya Mandala Surabaya Catholic University, Kalijudan 37, Surabaya 60114, Indonesia; valentinolunardi70@gmail.com (V.B.L.); felyciae@yahoo.com (F.E.S.); jindranyoo@yahoo.com (J.N.P.); shella_p5@yahoo.com (S.P.S.); mariayuliana@ukwms.ac.id (M.Y.); 2 Department of Chemical Engineering, National Taiwan University of Science and Technology, No. 43, Section 4, Keelung Rd, Da’an District, Taipei City 10607, Taiwan; 3 Research Centre for Sustainable Technologies, Faculty of Engineering, Computing and Science, Swinburne University of Technology, Kuching 93350, Sarawak, Malaysia; jsunarso@swinburne.edu.my; 4 Graduate Institute of Applied Science, National Taiwan University of Science and Technology, No. 43, Section 4, Keelung Rd, Da’an District, Taipei City 10607, Taiwan; yhju@mail.ntust.edu.tw; 5 Taiwan Building Technology Center, National Taiwan University of Science and Technology, No. 43, Section 4, Keelung Rd, Da’an District, Taipei City 10607, Taiwan

**Keywords:** drug delivery, drug release, functionalization, nanocellulose

## Abstract

The ‘Back-to-nature’ concept has currently been adopted intensively in various industries, especially the pharmaceutical industry. In the past few decades, the overuse of synthetic chemicals has caused severe damage to the environment and ecosystem. One class of natural materials developed to substitute artificial chemicals in the pharmaceutical industries is the natural polymers, including cellulose and its derivatives. The development of nanocelluloses as nanocarriers in drug delivery systems has reached an advanced stage. Cellulose nanofiber (CNF), nanocrystal cellulose (NCC), and bacterial nanocellulose (BC) are the most common nanocellulose used as nanocarriers in drug delivery systems. Modification and functionalization using various processes and chemicals have been carried out to increase the adsorption and drug delivery performance of nanocellulose. Nanocellulose may be attached to the drug by physical interaction or chemical functionalization for covalent drug binding. Current development of nanocarrier formulations such as surfactant nanocellulose, ultra-lightweight porous materials, hydrogel, polyelectrolytes, and inorganic hybridizations has advanced to enable the construction of stimuli-responsive and specific recognition characteristics. Thus, an opportunity has emerged to develop a new generation of nanocellulose-based carriers that can modulate the drug conveyance for diverse drug characteristics. This review provides insights into selecting appropriate nanocellulose-based hybrid materials and the available modification routes to achieve satisfactory carrier performance and briefly discusses the essential criteria to achieve high-quality nanocellulose.

## 1. Introduction

Drug delivery technology (DDT) is a cutting-edge applied science for delivering drugs to specific targets. This technology regulates the absorption and release of therapeutic drugs via various drug carriers to the desired organs, including subcellular organs, tissues, and cells, to improve human health [1]. DDT has advanced rapidly in the past few decades, enabled by various discoveries in various fields, including pharmaceutical, materials, and biomedical sciences. DDT development aims to improve therapeutic drugs’ pharmacological activity and overcome various disadvantages of conventional therapeutic drugs such as drug agglomeration, biodistribution deficiency, low bioavailability, limited solubility, and insufficient selectivity to prevent the concurrent effects of therapeutic drugs.

The majority of research studies on drug delivery technology revolve around developing materials suitable for drug delivery with desirable characteristics such as high drug adsorption capacity, targeted drug administration, controlled release, biocompatibility, and non-immunogenic and non-toxic effects that optimize therapeutic efficacy and eliminates side effects [2]. Many engineered nanomaterials have been studied for drug delivery applications [3]. Some nanomaterials have recently been undergoing development and clinical investigation; however, each nanomaterial has its various characteristics and limitations, challenging the researcher in creating a suitable drug delivery system.

Natural-based polymers have drawn considerable attention as suitable biomaterials for numerous applications in drug delivery systems. Various nature-based polymers such as polysaccharides (cellulose, chitosan, hyaluronic acid, pectins, alginate, cellulose ethers), proteins (silk fibroin and collagen), and peptides have been identified as promising biomaterials for drug delivery systems given their biocompatibility, processability, and characteristics (e.g., nanoparticles, hydrogels, aerogels, tablets, and so on) that can be regulated by modifying various polymer functional groups such as amino groups, carboxyl groups, and hydroxyl groups [4]. The current development of these mentioned various polysaccharides, proteins, and peptides for drug delivery systems have been well-reviewed elsewhere [4,5,6,7]. 

Several natural polymers have been shown to have a higher affinity for cell receptors and modulate cellular processes such as adhesion, migration, and proliferation. These advantages make these natural polymers attractive for effective and high-efficiency drug delivery systems [8]. They can also be degraded in the presence of in vivo enzymes, which ensures their ability to create responsive local delivery systems. However, only polysaccharides and proteins have been extensively studied in drug delivery systems (DDS). These natural polymers have unique characteristics in each tissue and have identical characteristics in the extracellular skeleton. These characteristics support these natural polymers’ utilization as drug carriers with insignificant invasive features [9,10,11].

Cellulose is the most abundant and commonly found natural polymer [12]. Its annual production is estimated at more than 7.5·10^10^ tons [13]. As a promising fuel and chemical precursor, cellulose has been widely utilized in various industries such as textile, pulp, paper, composite, and pharmaceutical excipients [2]. However, the development of cellulose-based materials as a direct molecule controller for drug adsorption and release had not been evaluated until the discovery of nanocellulose, which became a turning point for using carbohydrate-based nanomaterials in the field of drug delivery [14,15].

**Figure 1:**
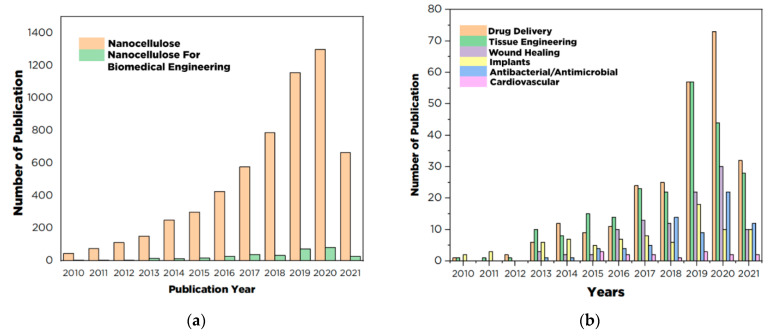
The number of publications in the area of nanocellulose and nanocellulose for biomedical engineering indexed by Scopus from 2010-until recent (10 June 2021) (**a**); data representation of annual publication of nanocellulose in various categories of biomedical engineering within the last decades (**b**); data analysis performed on Scopus using the terms nanocellulose and nanocellulose for “x” (x refer to biomedical engineering, drug delivery, tissue engineering, wound healing, implants, Antibacterial/antimicrobial, and cardiovascular).

As illustrated in Figure 1, the publication on nanocellulose for biomedical engineering applications increases every year, especially for drug delivery applications. The increase in the number of publications on the utilization of nanocellulose for drug delivery systems is a strong indication of the potential application of this material in the future. The rapid development of nanotechnology and materials science has brought about nanocellulose as a potential drug carrier because of its extraordinary physicochemical and biological characteristics. Nanocellulose has a large surface-area-to-volume ratio, thus enabling more significant adsorption and therapeutic drug-binding capacity than other materials. With these properties, nanocellulose can facilitate drug release mechanisms and allocate drug delivery precisely to the target to drastically reduce drug consumption, leading to improved drug delivery system effectiveness [16,17]. Nanocellulose additionally exhibits other attractive characteristics such as stiffness, high mechanical strength, biocompatibility, low toxicity, lightweight, tunable surface chemistry, and renewability [11,18], which are desirable for the design of advanced drug delivery system.

Nanocellulose can be utilized as either carrier or excipient for broad application in drug delivery systems such as microparticles, tablets, hydrogels, aerogels, regulating nanoparticles, and membrane drug delivery systems [19]. Nanocellulose has been manufactured on the laboratory and industrial scale, i.e., ranging from 140 g day^−1^ to 50 ton year^−1^ in three different forms as nanocrystalline cellulose (NCC), nanofiber cellulose (NFC), and bacterial nanocellulose (BNC) [20]. Several recent research and review articles have comprehensively overviewed the process, extraction, characterization, and applications of nanocellulose and their modified structures in drug delivery systems [12,17,21,22,23,24].

The drug binding and the release time of nanocellulose-based drugs vary depending on the nanocellulose configuration, therapeutical ingredient’s activity, the production method, and the modification [25,26]. Therefore, nanocellulose is a promising carrier for various drug delivery systems such as oral administration, ophthalmic drug delivery, intratumoral administration, transdermal drug delivery, topical administration, and local drug delivery.

This review provides a comprehensive overview of the preparation procedures of nanocellulose and the various effects on drug formulation and delivery. Three types of nanocelluloses and a brief description of their synthesis processes are discussed at the beginning of this review. Subsequently, the effects of raw materials and the synthesis process on the characteristics of the resultant nanocellulose are discussed. This is then followed by the application of nanocellulose to various drug delivery systems.

## 2. Conversion of Cellulose into Nanocellulose and Its Characteristic

Cellulose is the most abundant natural polymer globally and is a renewable source and essential raw material for various industries. Cellulose is a crucial constituent compound for plants, marine animals, algae, fungi, bacteria, and amoebae [12]. In 1838, French chemist Anselme Payen discovered and isolated cellulose from plant fibers using nitric acid and determined its chemical structure. The primary sources of cellulose are plant fibers with a high cellulose content, such as cotton (containing more than 90% cellulose content) [27] and wood (up to 50% cellulose). Other compounds such as hemicellulose, lignin, pectin, and wax are also present; they can be recovered during the separation process.

Recently, various agricultural wastes with high cellulose content were explored as a source of cellulose, such as oil palm empty fruit bunches (OPEFB) [28], palm and banana fronds, passionfruit peel waste [29], bagasse, wheat straw, rice straw, bamboo stalks, hemp bark, potato tubers, mulberry bark, hemp avicel, and sugar beets [30]. Cellulose derived from these non-plant precursors can have a molecular structure similar to that of plant cellulose. However, the main difference is that much less hemicellulose or lignin is present in these non-plant-based precursors; higher cellulose content with much lower impurities can be obtained from these precursors.

**Figure 2:**
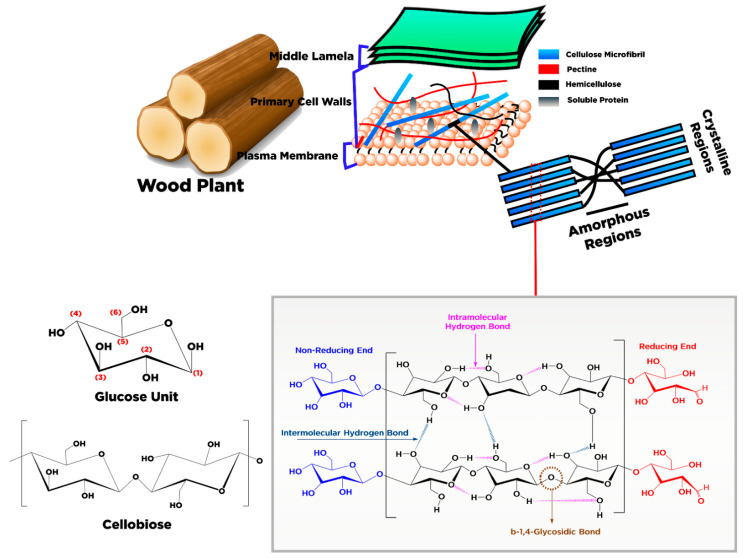
Schematic of cellulose production from wood plant and structural chemistry of exhibiting arrangement betwixt individual fibers.

In terms of chemical structure, cellulose is composed of a linear homopolysaccharide consist of β-d-glucopyranose units entirely condensed and bonded through β-1,4-glycoside linkages (Figure 2). The structure foundation of the cellulose network is arranged by a chain glucose dimer comprising two anhydrous glucoses (AG) defined as cellobiose [31] (Figure 2). The raw material or the pretreatment (chemical or mechanical) of cellulose may affect the cellulose chain length and thus lead to molecular weight variation. The number of AG units in each chain is known as the polymerization degree (PD). The value of PD for cellulose powder varies from 100 to 300 units and around 26,500 for cellulose pulp [32]. The PD value for cellulose from cotton is 15,000, and wood is approximately 10,000 [33].

Each cellulose monomer contains three reactive hydroxyl groups in the repeating chemical structure of the β-d-glucopyranose unit. In the same chain, these hydroxyl groups can make hydrogen bonds with the adjacent β-d-glucopyranose units. At different chain locations, the bonds present are intramolecular and intermolecular hydrogen bonds responsible for the crystal arrangement, determining the cellulose’s physical characteristics. Based on molecular orientation and hydrogen network between molecules and intramolecular, cellulose is classified into different types, i.e., I, II, III, III_I_, III_II_, IV_I,_ and IV_II_. For details about the classification of cellulose, the reader can refer to the work of Moon et al. [34]. Some of the cellulose characteristics are mainly represented by hydrogen linkage coordination [35,36].

Structurally, the cellulose is a linear chain polymer with a rod-like configuration, aided by the glucose residues’ equatorial conformation that is intensely aggregated together with the lateral size 3–5 nm [36]. Primary chains of cellulose, especially polysaccharide chains, are found on the secondary walls of plants arranged in a parallel configuration. The cellulose’s basic fibers have a cross-sectional diameter between 10–450 nm with a length of several micrometers that depend on the diversity of material sources [37]. Moreover, the elementary fibrils were arranged into large pack units called microfibrils, further foregathered into fibrils [13]. There are regions within the cellulose fibrils where the cellulose chains are organized into a highly crystalline structure with a length of 50–150 nm and disordered amorphous regions with 25–50 nm [34]. The cellulose chains construct the crystalline regions through Van der Waals forces, strong intra- and intermolecular hydrogen linkage, and β-1,4-glycosidic bonds. In contrast, amorphous regions are built up through the deficiency of hydrogen bonds in the crystalline region. The crystalline and amorphous regions in cellulose may vary depending on various sources.

**Figure 3:**
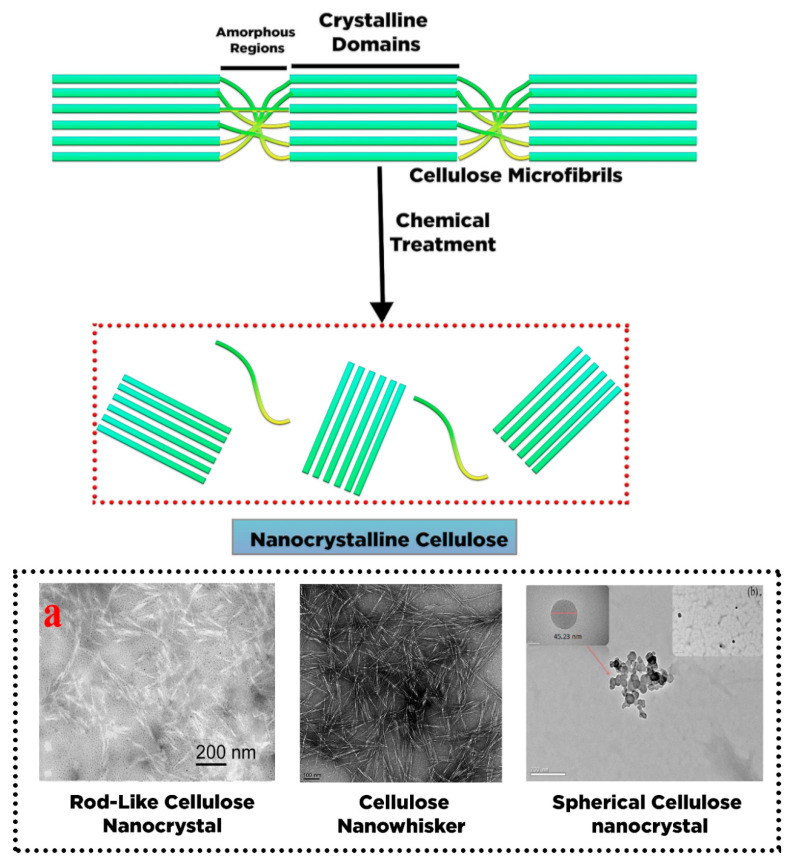
Schematic representation nanocrystalline cellulose fabrication by chemical treatment ((**a**) transmission electron microscopy (TEM) images of rod-like cellulose nanocrystals [38], reprinted with permission; transmission electron microscopy (TEM) images of cellulose nano whisker reprinted with permission from [25]. Copyright © 2019 Elsevier B.V.; (**b**) transmission electron microscopy (TEM) images of spherical cellulose nanocrystal reprinted with permission from [43]. Copyright © 2018 Elsevier B.V.).

The crystalline constituent within cellulose fibers can be refined through various chemical treatments by destructing and removing the disordered amorphous or para-crystalline regions. The purified crystal fragments with particle sizes on the nanometer scale are called nanocrystalline cellulose (NCC) (Figure 3). Different shapes of NCC are present such as needle and elongated rod-like shape or spindle-like shape with high stiffness of crystalline fragments [38], which are reported as cellulose whisker [39], nanowhisker [40], nanorod [41], and spherical nanocrystal [42].

A top-down process has been applied for NCC production in which a large unit of cellulose fibers (cm) is disintegrated through chemical or mechanical treatment into small units of nanocellulose (nm) [44]. NCC’s chemical structure is constructed by intra- and intermolecular hydrogen linkage of cellulose macromolecules with a high crystallinity value varying from 54 to 88% [45]. NCC’s particle size depends on the origin of the cellulose sources, with the diameter and length typically varying between 5 and 30 nm and between 100 and 500 nm, respectively [46]. Thus, NCCs have become an attractive candidate as drug carriers, given their outstanding physical and chemical properties [21,47,48].

**Figure 4:**
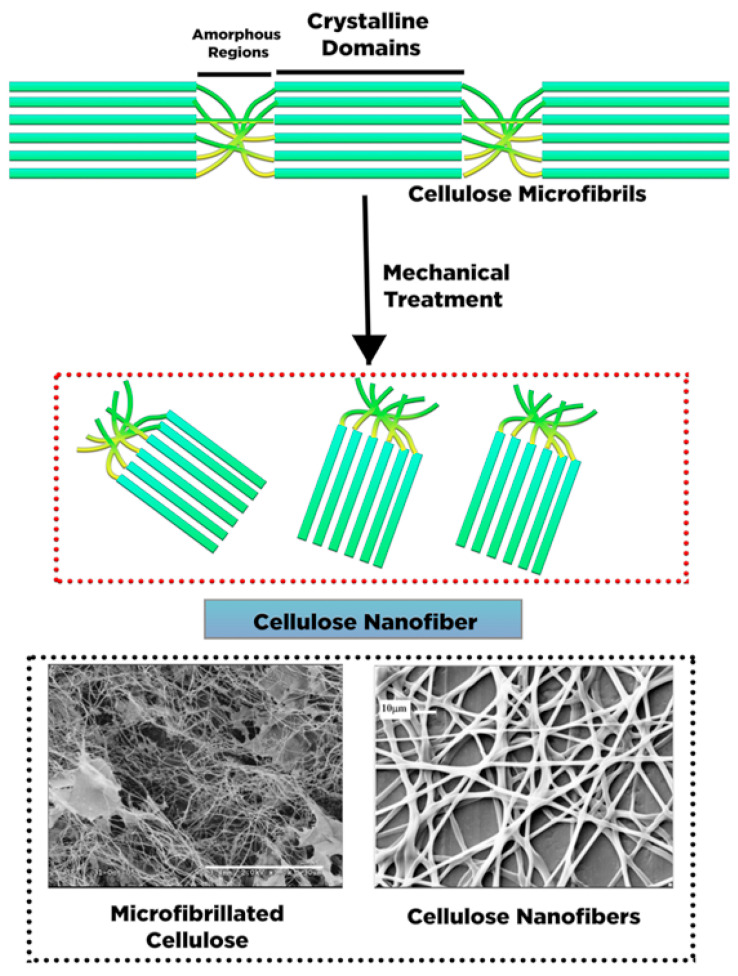
Schematic representation of cellulose nanofibers fabrication by mechanical treatment (scanning electron microscopy (SEM) images of micro fibrillated cellulose reprinted with permission from ref. [50]; Copyright © 2007 Elsevier Ltd.; scanning electron microscopy (SEM) images of cellulose nanofibers reprinted with permission from ref. [51]. Copyright © 2006 Elsevier Ltd.).

Cellulose nanofiber (CNF), also known as cellulose nanofibril, micro-fibrillated cellulose, nano-fibrillar cellulose, nano-fibrillated cellulose, or cellulose microfibril, has a similar molecule structure to NCCs with nano-size particles. Similar to NCC, CNF can also be produced from various cellulose sources. However, the morphology and crystallinity of NCC and CNF are the unique features that differentiate these two cellulose-based compounds. CNFs have long, flexible cellulose chains of amorphous and crystalline regions isolated from cellulose fibrils through mechanical treatment (Figure 4) [46]. The diameter of CNFs varies from 1 to 100 nm, while their length varies between 500 and 2000 nm. The dimension of CNFs molecules is strongly influenced by mechanical treatment and defibrillation [49].

NCC has high crystalline cellulose purity, resulting in a rigid structure, whereas the CNF structure consists of irregular amorphous parts, with some parts exhibiting a high degree of crystallinity. The amorphous regions in CNF control the structure flexibility of nanocellulose [52]. Figure 4 presents an illustration of CNF extracted from cellulose fragments via mechanical defibrillation. The exerted force fractures the cellulose fibrils along its longitudinal axis [34]. Compared with NCC, CNF exhibits unique properties such as extended length with excellent aspect proportion (length to diameter), superlative surface area, hydrophilicity, biocompatibility, and adjustable characteristic through surface modification [53].

**Figure 5:**
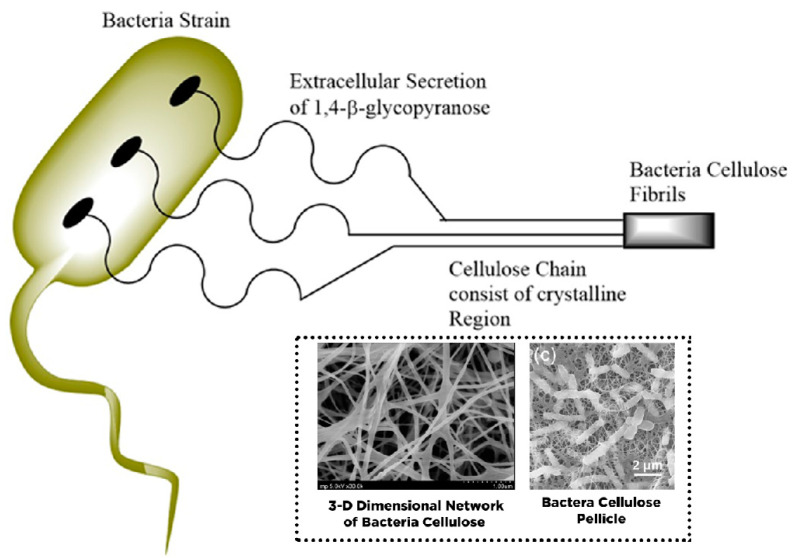
Schematic production of bacteria cellulose through extracellular secretion (scanning electron microscopy (SEM) images of 3-D dimensional network of bacteria cellulose [58], reprinted with permission; (**c**) scanning electron microscopy (TEM) images bacteria cellulose pellicle, reprinted with permission from ref. [59]. Copyright © 2019 Elsevier Ltd.)

Microbial cellulose (MC), bacterial nanocellulose (BC), and bio-cellulose (BC) have been used as the other terms for bacteria cellulose (BC). In contrast to NCC and CNF, BC’s structure comprises sugars with low molecular weight. Many bacteria strains have been used to generate BC as an extracellular metabolic product, such as *Gluconacetobacter, Sarcina, Aerobacteria, Escheria, Achromobacter, Rhizobium, Rhodobacter, Azotobacter,* and *Agrobacterium* [54,55]. However, only *Gluconacetobacter xylinus* has been commercially utilized to produce BC on an industrial scale [27]. The bacteria strains are commonly incubated in nutrient-rich aqueous media and produce BC on the upper layer (interface with air) as an exopolysaccharide. In this case, the β-d-glucopyranose units are initially present during the growth of cellulose molecules within the bacterial cell. The elementary fibril is released across the pores of the cellulose surface, which was further arranged and crystallized into microfibrils with twisting ribbons shape followed by pellicle formation (Figure 5) [56]. The fabricated BC comprises a nanofibers framework with a diameter of 20–100 nm with a length of several micrometers and a large surface area composed mainly of water (99%) [57].

In terms of chemical composition, BC is indistinguishable from plant-based nanocellulose (e.g., NCC and CNF). However, BC has higher crystallinity (up to 84–89%) with fewer amorphous regions than NCC and CNF. Moreover, BC contains fewer impurities and contaminants such as hemicellulose, lignin, and pectin, mainly found in plant-based nanocellulose. BC is a biocompatible material with non-cytotoxicity and non-genotoxicity for biomedical applications, especially drug delivery [60]. BC synthesis does not involve a complicated process such as mechanical and chemical treatment to cleave the hemicellulose or lignin within the lignocellulosic biomass, thereby allowing high cellulose purity.

BC’s properties can be modulated by various techniques such as substrate manipulation, culture condition and operation parameter, and proper bacterial strain selection [17,54]. In contrast to NCC and CNF, BC provides unique characteristics such as high crystallinity of nanocellulose (84–88%) and polymerization grade, high water uptake capacity (exceeding 100 times of its weight), large surface area (high aspect proportion of fiber), outstanding tensile strength (Young modulus of 15–18 GPa), flexibility, foldability, moldability, mechanical stability, and high porosity [60]. A summary of the characteristics of various types of nanocellulose is listed in Table 1.

Based on the previous discussion, cellulose can be subjected to a mechanical, biological, and chemical treatment to produce three different NCs, i.e., nanocrystalline cellulose, cellulose nanofibrils, and biological cellulose. They are classified based on various aspects such as morphology, particle size, crystallinity, nanocellulose structure, extraction techniques, and cellulose sources [56]. Moreover, other important factors such as interfibrillar arrangement, microfibril inclination, chemical constituent, cell dimension, and defects can also vary depending on the cellulose sources [62]. Among all the mentioned characteristics, mechanical strength is essential in the drug delivery field [63]. As summarized in Table 1, NCC possesses a high modulus young, up to 220 GPa, which is higher than glass (86 GPa) [61] and kevlar KM2 fiber (88 GPa) [45]. Furthermore, the mechanical stiffness of NCC can reach up to 7.7 GPa, which is higher than 302 stainless steel (3.88 GPa) [45] and kevlar KM2 fiber (1.28 Gpa) [45].

## 3. Sources and Pretreatment of Raw Materials for Nanocellulose Productions

In general, the production of nanocellulose (NC) consists of three steps: (1) Finding the suitable sources, (2) raw material pretreatment, and (3) NC extraction. The raw material’s source and type influence the physical and chemical properties and the NC product’s yield. Currently, most nanocellulose sources utilize high-quality biomass such as cotton, wood pulp, and dissolving pulp, which comprises the high cellulose content. However, in response to recent essential issues, such as the depletion of non-renewable energy and increasing global temperature, the researchers realized the development of waste-based biomass as a feedstock for the production of nanocellulose. Various types of biomass waste, including forest residues, algae, agricultural, and industrial by-products, appear as potential raw materials for nanocellulose production. In terms of chemical composition, each category of biomass waste is primarily composed of cellulose, lignin, hemicellulose, pectin, and other minor substances with different physical and chemical characteristics [64]. Agricultural and forest residues have similarities in their chemical composition, but lignin composition in agricultural waste is significantly high, while the cellulose content in forest residues is higher than in agricultural waste [64,65]. 

Among all of the waste-based cellulose sources, the nanocellulose extraction from industrial waste seems more complex since the chemical and structural composition of feedstock is variable and crucially depends on the residue types. The various impurities (e.g., hemicellulose, lignin, wax, and pectin) act as a structural barrier that hinders the accessibility to the cellulose material for the extraction process [22]. Therefore, pretreatment is necessary to remove the cellulose framework’s impurities, permitting the aperture of the material framework to expedite cellulose microstructure access. Moreover, removing impurities is also beneficial to reduce the consumption energy of mechanical treatment for cellulose disintegration [66]. Another objective of raw material pretreatment is to regulate the biomass structure and size and overcome the plant cell wall recalcitrance.

The pretreatment is generally divided into four categories such as physical (milling, grinding, microwave, ultrasound, etc.), chemical (dilute acid, mild alkali, TEMPO mediated oxidation, organosolv, and ionic liquid), biological (fungi, bacterial, and archaeal), and physicochemical (steam explosion, liquid hot water, wet oxidation, etc.) [67]. The effectiveness of the biomass pretreatment process depends on pH, temperature, type of catalyst, and pretreatment time. Selecting the appropriate pretreatment would allow avoiding the structure disintegration or loss of cellulose, ensuring low cost, and minimizing energy use to reduce toxic and hazardous waste [68].

The chemical pretreatment process is considered the most efficient and economically feasible for the disintegration of biomass with low pretreatment severity. However, chemical pretreatment is non-environmentally friendly and requires a wastewater treatment process [69]. Physical pretreatment is environmentally friendly and scarcely generates hazardous or toxic substances, but the major disadvantage lies in its high energy consumption, which is generally higher than chemical treatment [70]. Biological treatment is widely known as an eco-friendly process, operates under mild conditions, and consumes a lower energy amount. However, long pretreatment duration, low conversion, and carbohydrate loss tendency throughout pretreatment remain the main challenges of biological pretreatment by the microorganism [71]. Physicochemical pretreatment using a combination of chemicals and high temperature or pressure in extreme conditions can effectively escalate biomass degradation. Nevertheless, high energy input is required, which translates to high operation costs for this method. Proper pretreatment of cellulosic fibers can improve the hydroxyl group’s accessibility, inner surface enhancement, crystallinity alteration, and fracture of the intra and inter hydrogen bonds of cellulose, leading to the increased fibers reactivity [72]. Detailed pretreatment of cellulose-based raw materials has been thoroughly discussed elsewhere [73]. 

The integrated pretreatment strategy of lignocellulosic waste biomass comprising two or more pretreatment stages increases the pretreatment process’s effectiveness, product characteristics, and versatility of composition in extracted cellulose. An additional process that adds more steps to cellulose purification is highly undesirable [74]. For instance, de Carvalho Benini [75] performed alkaline treatment coupled with multiple stages of bleaching pretreatment followed by sequential dilute acid hydrolysis to increase the efficiency of impurities removal (e.g., starch, hemicellulose, and lignin/pectins) from the cellulose framework. Similarly, Wijaya et al. [29] combined alkaline and bleaching treatment to obtain higher purified cellulose from passion fruit peel. In a different study, Maciel et al. [76] obtained the soluble and insoluble lignin after alkaline treatment reached 60 and 75%, respectively. The summary of the pre-treatment strategy of waste-based nanocellulose sources is presented in Table 2. 

## 4. Isolation of Nanocellulose 

### 4.1. Isolation of Nano-Fibrillated Cellulose (NFC)

Regardless of its cellulose sources, NFC is mainly fabricated from cellulose pulp through mechanical treatment by breaking down the linkage of interfibrillar hydrogen [106]. The exerted mechanical force triggers the cracking phenomenon to form a critical tension center in fibrous substances. The development of NFC from fibrous material requires intense mechanical treatment with or without pretreatment. However, fibrous material’s mechanical disintegration may cause pulp clogging, causing the fiber to agglomerate and require high energy to break it down. Thus, another pretreatment is required to overcome this problem.

Several pretreatments have been introduced before the primary mechanical treatment to diminish the polymerization degree and debilitate the hydrogen linkage. These pretreatments include mechanical refining, alkaline hydrolysis, solvent-assisted pretreatment, organic acid hydrolysis, 2,6,6-tetramethylpiperidine-1-oxyl (TEMPO)-mediated oxidation, enzymatic disintegration, periodate-chlorite oxidation, oxidative sulfonation, cationization, ionic liquid, carboxymethylation, deep eutectic solvents, and acetylation [17].

The earliest production of NFC was reported by Turbak et al. [107] and Herrick et al. [108]. They isolated NFC from wood via high-pressure homogenization (HPH). HPH exerted a mechanical force on cellulose fibrils driven by crushing, shear, and cavitational forces in which cellulose pulp is transferred into the chamber through a small nozzle to enable particle size reduction to the nanoscale of the cellulose fibrils [72]. Currently, the HPH is the most commonly utilized method for NFC production on an industrial and laboratory scale, given its simplicity, high efficiency, and lack of organic solvent requirements [109]. Furthermore, HPH enables high conversion of cellulose material toward CNF. High energy, high pressure, and long duration of the HPH process may also escalate the fibrillation degree. However, the difficulty of cleaning the equipment due to the blockage in the homogenizer valve is the major drawback of the HPH method [110]. Different processes have also been developed to produce CNF, such as micro-fluidization, micro-grinding, cryo-crushing, ultrasonication, mechanical refining, radiation, ball milling, blending, extrusion, steam explosion, and aqueous counter collision [111].

### 4.2. Isolation of Cellulose Nanocrystal (NCC)

According to the previous discussion, the main difference between NCC and CNF lies in their structure, in which CNF comprises amorphous and crystalline regions while NCC has high crystalline purity in cellulose regions. Therefore, the primary step in isolating NCC is to break down the disordered amorphous or paracrystalline regions that integrate the crystalline regions within cellulose fibrils. Initially, an NCC suspension was produced in 1949 from lignocellulosic biomass through an integrated alkaline and bleaching pretreatment and acid hydrolysis [13]. Acid hydrolysis remains the paramount process for NCC extraction. The crystalline part in cellulose fibers is not hydrolyzed because it has a high resistance to acids, although acids can easily hydrolyze the amorphous regions [112]. In this method, sulfuric acid (H_2_SO_4_), hydrochloric acid (HCl), hydrobromic acid (HBr), and phosphoric acid (H_3_PO_4_) have been extensively employed as the acid component to breakdown the amorphous region of cellulose.

Following acid hydrolysis, the remaining free acid molecules and other impurities should be removed by diluting and washing with water using centrifugation and dialysis processes. Moreover, specific mechanical treatment like sonication may be needed to stabilize the NCC particles in uniform suspensions. However, the high tendency of corrosion, low recuperation rate, and high acid wastewater produced due to the high amount of water for the washing process for nanocellulose suspension neutralization become the significant drawbacks of the acid hydrolysis process [46]. To avoid excessive equipment corrosion and environmental issue, various nanocellulose isolation processes have been developed, such as extraction using ionic liquids, TEMPO oxidation, enzymatic, and others. Various researchers have carried out the combination and integration of various isolation processes to increase the isolation process’s efficiency, such as enzymatic hydrolysis with TEMPO oxidation and enzymatic hydrolysis with ultrasonication [113]. Chemical treatment is crucial for NCC isolation, while mechanical treatment is the vital stage for CNF production.

### 4.3. Isolation of Bacteria Cellulose (BC)

The selection of strains of microorganisms is a very crucial factor in the synthesis of BC. There are currently two main methods that have been used for BC production, i.e., static fermentation and submerged fermentation [54]. Static fermentation has been widely employed as an extracellular-based production route. In the static fermentation, a 3D network of gelatinous pellicles with high water content formed during the interspersing and intertwining of the ribbons structure form of BC, reaching a particular thickness corresponding to longer incubation time and causing the entrapment of bacteria cells and its further inactivity. The static fermentation produces BC with excellent crystallinity and mechanical strength, although prolonged cultivation and low productivity limit their industrial utilization.

Furthermore, the BC layer’s uneven thickness is produced due to the exposure of bacteria to uncertain conditions (nutrient, oxygen level, and cell distribution) throughout the growth cycle. Fed-batch strategies and submerged fermentation involving aeration and agitation fermentation have been introduced to overcome static fermentation’s significant drawbacks. Submerged fermentation leads to higher BC productivity than static fermentation, which has been extensively utilized commercially. The cultivated bacteria are adequately exposed to oxygen, thereby generating a high yield of BC in the shape of small granules or pellets during aerated fermentation [114]. Moreover, agitation in the fermentation would result in a more homogeneous BC and oxygen evenly distributed to bacterial cells. However, the produced BC has lower crystallinity and mechanical strength than static fermentation [115].

Several submerged fermentation issues such as the advancement of cellulose non-production strains [116], irregular shapes of BC granules or pellets, and physical characteristic modification of BC remain challenging for the researcher to overcome. In addition, excessive-high rotation speed and hydrostatic stresses may promote gluconic acid production by bacteria due to the accumulation of self-protection metabolism [117]. Several factors such as bacterial strains, fermentation medium carbon sources, growth condition, and its characteristic and yield should be evaluated carefully to choose the most suitable BC synthesis process selection approach. The summary of the recent studies of BC production is given in Table 3.

## 5. Surface Chemistry of Nanocellulose for Drug Delivery 

Biocompatibility, biodegradability, and drug carrier capability to confine, control, and localize the drug release towards the target sites are desirable for nano-drug carrier formulation. The ability of nano-drug carriers to transport the drug and specify the sites for targeted drug release is influenced by the particle size, the surface charge, modification, and hydrophobicity. These aspects govern the nano-drug carrier interface with the plasma membrane and its diffusion across the physiological drug barrier [123]. Most NCs exhibit high specific surface area and negative interface charges as potential drug carriers, making them suitable as hydrophilic drug carriers. Therefore, the NCs’ surface can be attached to the desired drug [124]. However, pristine NC cannot be used effectively as a drug carrier given its limited water solubility, moisture sensitivity, thermal instability, and lack of stability in various buffer solutions. Even though the pH adjustment of the environment can enhance the dispersibility of NCs, the scattering examination divulged the aggregation tendency of NCs, which translates to the colloidal instability of NCs. The size reduction obtained by converting cellulose into NC provides an exponential improvement of hydrogen bonding that triggers the NC aggregation. This limitation can be made worse by the drug coordination, which is exposed on the NC exterior, consequently altering the dispersibility and solubility [125]. Therefore, various surface modification and pretreatment fiber methodologies have been developed to overcome limitations and advance specific characteristics [126].

From a structural perspective, the three hydroxyl groups in each cellulose monomer are the most prominent characteristic that makes the NC surface reactive. The reactivity of hydroxyl groups influences the surface modification of anhydroglucose units. It was reported that in the molecular framework of cellulose, the hydroxyl group at the sixth position behaves as primary alcohol with a reactivity ten times larger than the other hydroxyl groups, while the hydroxyl group at the second position has two-fold higher reactivity than that in the third position, both of which serve as secondary alcohols. This phenomenon manifests from the steric hindrance of each hydroxyl group, in which the hydroxyl group at the sixth position attached to the carbon atom that is connected to only one alkyl groups while the carbon atom that carries the hydroxyl groups in the second and third positions bonded to two alkyl groups [127]. Regarding the surface receptiveness of NC’s hydroxyl groups, the addition of solvent and reactant may alter the group’s receptiveness in diverse positions. De la Motte et al. [128] modified NCC through cationic epoxide 2,3-epoxypropyltrimethyl ammonium chloride (EPTMAC) by spray technique. It was revealed that the hydroxyl bunch receptiveness of cationic modified NC follows the order of OH-C_6_ = OH-C_2_ > OH-C_3,_ which was validated through nuclear magnetic resonance (NMR).

Nanocellulose surface modification for drug delivery was developed by modulating the NC hydroxyl groups. In general, the main objective of nanocellulose surface modification is to incorporate new functional groups or drug components into the nanocellulose framework to escalate the degree of substitution and the efficacy of material grafting without altering the structure, morphology, and crystallinity of nanocellulose [129]. Several processes have been developed for the surface modification of NC, either by physical or chemical processes, presented in more detail in the following sections.

### 5.1. Functionalization of Nanocellulose through Physical Technique

Several physical techniques such as surface defibrillation, irradiation, electric current, and electric discharge have been developed to modify and functionalize nanocellulose surfaces for diverse applications [130]. Surface defibrillation disintegrates cellulose into elementary fibrils by exerting mechanical force using various devices such as ultra-refining, a high-pressure homogenizer, a grinder, a microfluidizer, and spray-drying. In nanocellulose functionalization, the combination of nanocellulose and drug entities can be subjected to surface defibrillation to modify the morphology of nanocellulose and construct a new matrix system with a tight fiber network.

Microparticles from BC with fibrillar structure morphology have been prepared by spray-drying technique. An ultra-refining-assisted method was also conducted to construct bacteria cellulose nanofiber (BCNF) with various sizes and shapes. The coating of BCNF with mannitol (MN), maltodextrin (MF), and hydroxypropylmethylcellulose (HPCM) were also carried out at various ratios to study the drug release characteristics. The addition of such coating matrices exhibits benefits towards the spray-drying process and drug carrier ability, i.e., superior protection of drug confinement, decreased droplet adhesion on the drying chamber, and improved powder performance. As a result, the BC-microparticles can successfully enhance the drug uptake capacity and sustain the drug release of diclofenac sodium (hydrophilic) and caffeine (lipophilic) [131]. 

As a recent advanced method, irradiation exerted high energy, which modifies the cellulose exterior. For example, the radiated gamma energy can generate reactive intermediates comprising ions and free radicals that provoke reaction pathways such as cross-linking, scission degradation, oxidation, and polymer and molecule grafting. The presence of irradiation beams, such as microwave and electron, accelerates the polymer growth. UV-irradiation has also been developed to improve the reaction rate to allow pre-synthesized grafted polymer formation on the nanocellulose surface [132]. Recently, this method has been developed to induce polymer grafting and polymer growth on nanocellulose surfaces.

Plasma treatment is considered an environmentally friendly method to achieve nanocellulose surface functionalization by utilizing plasma ionized gas without altering its characteristics. Researchers have widely applied this method for various modifications, such as increasing material–cell interaction, introducing the surface of NC with hydrophobicity or hydrophilicity characteristics, and incorporating chitosan towards cellulose substrates. For instance, Kusano et al. [133] modified CNF by utilizing dielectric-based plasma discharge treatment, leading to the formation of many carboxyl groups, carbonyl groups, and oxygen-containing groups on the surface of nanocellulose [133]. Moreover, assisted ultrasonic irradiation combined with plasma discharge treatment can refine the wetting and oxidation of the nanofibers coating. Plasma treatment is an attractive route for surface functionalization of nanocellulose given its benefits such as non-polluting, fast-modification, and simple chemical treatments compared to the conventional modification method.

### 5.2. Functionalization through Chemical Synthesis of Nanocellulose

**Figure 6:**
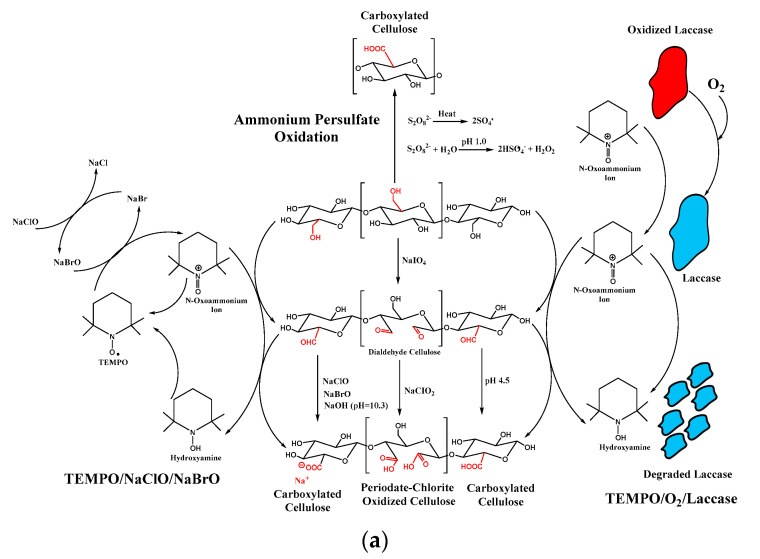

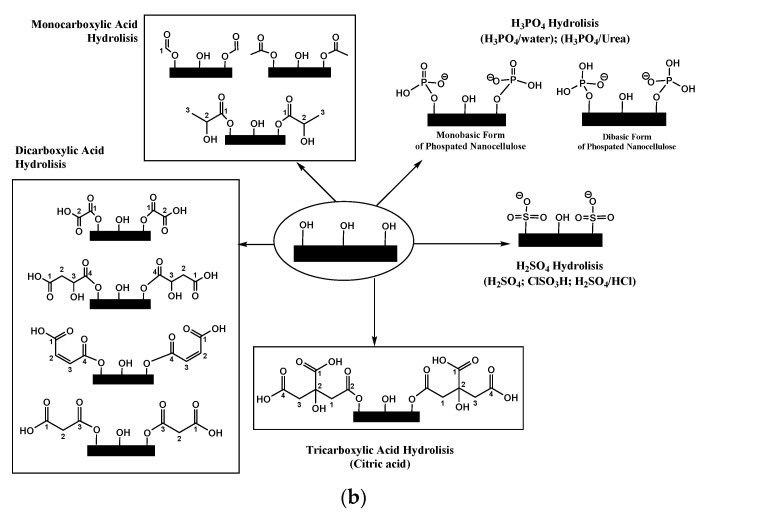
Simplified mechanisms of chemical synthesis nanocellulose; (**a**) acid-based chemical modification; (**b**) oxidation based chemical modification.

Chemical treatments use reactive chemical species for nanocellulose formation through cellulosic framework disintegration. As mentioned in the previous section, acid hydrolysis has been extensively exploited as the primary process for CNF and NCC isolation from the cellulosic fiber. The strong acidic environment leads to the disintegration of amorphous regions that act as structural defects in the cellulosic framework, facilitating nanoparticle production. Other chemical processes, such as TEMPO-based oxidation and APS oxidation, are also used in the CNF and NCC synthesis. The schematic mechanisms of acid-based hydrolysis and oxidation processes are presented in Figure 6. The summary of chemical modification of nanocellulose is tabulated in Table 4.

In general, NCC isolation comprises exposing pure cellulose material under strong acid hydrolysis with strictly controlled operating parameters such as temperature, agitation, time, and concentration of chemical species. As mentioned earlier, various chemical reagents such as H_2_SO_4_, HCl, HBr, and H_3_PO_4_ have been utilized as cellulosic disintegrators. The selection of acid reagents has the most crucial role in determining drug carrier characteristics and synthesis pathways for incorporation or grafting through chemical/physical modification for particular functional groups. The amorphous decomposition using HCl and HBr is not widely adopted because they provide low dispersion stability of NCC and increase the agglomeration tendency of NCC in an aqueous suspension. H_2_SO_4_ and H_3_PO_4_, on the other hand, exhibit better performance as a hydrolyzing agent because the chemical moieties can be attached to the hydroxyl group of NCC during the reaction to isolate charged surface of NCC for subsequent incorporation of phosphate or sulfate functional groups. The new functional group incorporation causes the spontaneous dispersibility of NCCs in an aqueous environment due to the colloidal stability restoration through electrostatic repulsion refinement, which is the preferred characteristic of drug carriers.

A subsequent treatment of H_2_SO_4_ followed by HCl synthesis has been utilized to control the sulfate moieties on the NC surfaces. The as-synthesized particle had a similar particle size to those particles directly acquired from acid hydrolysis. Nevertheless, the surface charge density can be adjusted on the hydroxyl groups exploited by sulfate groups [49]. Lin and Dufresne [137] proposed a strategy of inaugurating progressive sulfate group content on NCCs surface through the modulation ratio of reactants and post-sulfonation (chlorosulfonic acid) and desulfonation conditions. They also evaluated the impact of sulfonation degree on the morphology, dimension, physical characteristic, and surface chemistry of modified NCCs. Diverse zeta potential ranged from −7 mV to −66 mV and approximately 0.0563 mmol/g–1.554 mol/g of sulfonation degree was acquired. Therefore, it is indicated that the zeta potential of nanocellulose is mainly controlled by the sulfonation degree of nanocellulose itself [137]. 

Wijaya et al. [29] successfully isolated NCC through sulfuric acid hydrolysis of bleached passionfruit peels waste fiber by adjusting the acid concentration, hydrolysis time, and reaction temperature. The NCC was used for tetracycline hydrochloride adsorption through electrostatic and Van der Waals interaction. The adsorption isotherm was correlated using Langmuir and Freundlich isotherm models. With pH environment adjustment, the adsorption affinity of the drug can be altered to control the uptake and sustained release of drugs [29].

(2,2,6,6-tetramethylpiperidine-1-oxyl)-mediated (or TEMPO-mediated) oxidation of nanocellulose has arisen as an alternative NC isolation route to replace the conventional acid hydrolysis method due to its environmentally friendly and facile synthesis nature. The synthesis starts by using TEMPO/NaBr/NaClO or TEMPO/NaClO_2_/NaClO as a reagent. TEMPO (stable nitroxyl radical) forms as the catalyst for NC synthesis, which further transforms into *N*-oxoammonium salt (R_1_R_2_N^+^=O) under certain conditions while the NaClO acts as a primary oxidant [46]. Both NaClO and NaBr can reversibly transform the *N*-oxoammonium salt into TEMPO form. The hydroxymethyl groups of NC (primary hydroxyl groups located on C6) are selectively transformed into carboxylated groups while the secondary hydroxyl groups remain unchanged (secondary hydroxy groups located on C2 and C3) [66]. The incorporated carboxyl groups imparted negative surface charges from the change in the environment pH, which leads to improved colloidal stability. 

As reported by Montanari et al. [154], TEMPO-mediated oxidation with the degree of oxidation 0.24 has imparted negative charges on the crystalline regions of nanocellulose, which provide dispersibility and individualization improvement time decreasing the crystallite size [154]. Meanwhile, Habibi et al. [150] underlined that the TEMPO-mediated oxidation did not affect the morphological and crystallinity of NCCs. Furthermore, they highlighted that the ratio of primary oxidizing agents affected the negative charge of NCCs [150]. 

A novel oxidation system of TEMPO/laccase/O_2_ has been utilized to modify NC. The TEMPO/laccase/O_2_ system with sufficient catalytic amounts of laccase and TEMPO reagent produced reactive TEMPO^+,^ which subsequently transformed primary hydroxyl moieties into aldehyde moieties through oxidation. After the oxidation, the reactive TEMPO^+^ was reduced into *N*-hydroxyl TEMPO. However, no-cycle regeneration occurred between TEMPO^+^ and *N*-hydroxyl TEMPO due to the breakdown of the primary hydroxyl groups of polysaccharides and laccase molecules. Furthermore, the *N*-hydroxyl-TEMPO was accumulated in the reaction environment due to the absence of active laccase in the system. Therefore, a large amount of TEMPO and laccase and prolonged reaction time are required to oxidize the primary hydroxyl groups, which are considered major disadvantages of this process [151]. 

TEMPO-mediated oxidation was mainly used to modify NFCs before mechanical defibrillation to promote the fiber’s individualization. TEMPO-mediated oxidation leads to the breakage of the strong intra-fiber hydrogen coordination to facilitate the softening and impairing of its rigid structure, which is beneficial in converting TEMPO-oxidized cellulose fiber into highly crystalline individual nanofibers through mechanical treatment. The NaClO concentration and mechanical treatment strength were considered crucial factors in determining the polymerization degree, carboxylate group numbers, and CNFs yield.

Carlsson et al. [155] emphasized the influence of surface charges in nanocellulose formulation as a drug carrier by introducing TEMPO-mediated oxidation in mesoporous claodophora cellulose for aspirin degradation. The surface charge negativity (carboxylate content 0.44 ± 0.01 mmol g^−1^) significantly accelerated the degradation of aspirin compared to the native source of CNFs, which had a deficient surface charge (0.06 ± 0.01 mmol g^−1^). This phenomenon is caused by the strong interaction of opposite charge entities between aspirin and TEMPO-oxidized cellulose nanofibers (TOCNFs), leading to increased partial amorphization ability inside the mesoporous TOCNFs [155].

Without a chlorine-containing oxidant, 1.1 mmol g^−1^ of carboxyl groups were incorporated onto wood cellulose. High in carboxylate content, wood cellulose underwent tremendous depolymerization during oxidation. In addition, a long reaction duration of up to 15 h was required to achieve 0.6 mmol g^−1^ carboxylate content, while 1.1 mmol g^−1^ was achieved by increasing the reaction time up to 20 h. Prolonged reaction time is considered the major disadvantage of this process. This method has been utilized for nanocellulose modification in drug delivery applications [156]. The sequential periodate-chlorine oxidation selectively and simultaneously incorporates two carboxyl groups through the oxidative transformation of two vicinal secondary hydroxyl groups (located in C2 and C3 instead of C6 position), enabling higher surface charge density introduction. The increase of surface charge density is essential in retaining the colloidal stability of drug carrier and improving the electrostatic interaction between drug and carrier, which increase the loading uptake of drugs. 

Plappert et al. [152] investigated the pretreatment effect of sequential chlorite periodic oxidation on open-porous anisotropic CNF hydrogel membrane assembly. Hydrogel membranes were used for transdermal drug delivery systems for nonsteroidal anti-inflammatory drugs (NSAIDs) and piroxicam (PRX). By tuning the surface charge density and the amount of carboxylated groups (0.74–2.00 mmol g^−1^) by varying the reagent concentration, the drug carrier uptake capacity can be increased to within the range of 30–60 mg g^−1^ with the surface charge −66 mV to −128 mV. The electrostatic interaction between the cationic drug (PCX) and the anionic characterized surface of CNF membranes is the main driving factor behind the loading of drugs in the membrane [152].

### 5.3. Functionalization through Post Chemical Modification via Covalent and Physical Bonding Strategy 

Maintaining the structural integrity of nanocellulose to prevent the polymorphic transformation and maintaining the crystalline area while modifying its surface are considered the main challenges. Therefore, several post-chemical modifications have been studied for surface modification and functionalization of nanocellulose surfaces before the drug upload. Sulfonation treatment is the most common strategy to introduce sulfate groups into hydroxyl moieties of nanocellulose, which produces a highly negatively charged surface. Nevertheless, the degree of sulfonation was highly determined by several factors such as temperature, acid concentration, and hydrolysis time. Treatment of NC with sulfuric acid or sulfonation followed by acid hydrolysis [137,157] can improve the characteristics of NCs. However, these improvements may lead to lowering the colloidal stability of NC due to the reduction in the sulfonate degree. Since the primary goal of the drug delivery system is to achieve higher colloidal stability and strong electronegativity for further electrostatic drug adsorption or modification, straight H_2_SO_4_ hydrolysis remains the primary treatment for NC modification.

On account of the simple and straightforward treatment, modification of hydroxyl groups at the NC surface by Fischer esterification is another common approach. Several reactants have been used to acetylate the surface of nanocellulose, such as acetic, citric, malonic, and malic acid with the combination of HCl or H_2_SO_4_. The utilization of H_3_PO_4_ provides NC modification with higher thermal stability than sulfonated NC. Camarero Espinosa et al. [135] suggested that only one hydroxyl group was incorporated by one ester bond of phosphoric groups. Another study by Kokol et al. [138] revealed the possibility of phosphate-modified nanocellulose (P-NC) originating from two structural isomers, either of which can behave as monobasic acid or dibasic groups. Acetylation of hydroxyl groups of NC can also be performed using enzymatic modification. In an environmentally friendly approach, enzymatic modification serves as a favorable modification route without the need for any addition of chemicals and has low energy requirements, improving biocompatibility and lowering the cytotoxicity of NC for drug delivery.

The acid hydrolysis and oxidation treatments are mainly considered as a primary synthesis of nanocellulose. Indeed, during acid-based hydrolysis or TEMPO-oxidation, hydroxyl groups of nanocellulose grafted by anionic sulfate ester groups (-OSO_3_^−^) and carboxylate groups (-COOH) produce the negative electrostatic layer of nanocellulose. Consequently, high stability of nanocellulose occurs in the aqueous solution resulting in electrostatic repulsion between individual particles. Maintaining the structural integrity of nanocellulose to prevent the polymorphic transformation and maintaining the crystalline area while modifying its surface are considered the main challenges. Several post-chemical modifications have been studied for surface modification and functionalization of nanocellulose surfaces before the drug upload. 

Silylation is another approach to modify the surface nanocellulose by conjugating small molecules. A series of alkyl dimethyl-dimethylchlorosilane (alkyl-DMSiCl) with various alkyl groups such as isopropyl, *N*-butyl, *N*-octyl, and *N*-dodecyl can be grafted on the surface of NCC in the presence of toluene. However, the high price and high toxicity of the reagents limit the progress of silylation modification in the drug delivery field. Recently, Li et al. [158] developed an NC template for mesoporous hollow silica material (R-nCHMSNs) for ibuprofen and lysozyme drug delivery. The presence of NC as a template increases the content of geminal silanols on the R-nCHMSNs surface. Nanoparticles with high content of geminal silanols present outstanding delivery characteristics for various drugs [158].

The amine derivatives can covalently bond the surface of NC through a carbodiimide amidation reaction. The majority of amidation-mediated couplings were incorporated on the carboxylic groups of pre-oxidized NC without re-molding the morphology and crystalline native structure. *N*-ethyl-*N*-(3-dimethylaminopropyl) carbodiimide hydrochloride (EDAC) has been widely used for the amidation among carbodiimide derivatives. The addition of *n*-hydroxysuccinimide (NHS) is required to avoid unstable intermediate O-acyl urea formation and to achieve the direct formation of the stable *N*-acyl urea. The amidation approach was presented by Akhlagi et al. [159] to create a drug delivery system based on chitosan oligosaccharides (CSOS) and TEMPO-oxidized NCC. The carboxylic moieties on the oxidized NCC were coordinated into the primary alcohol and amino moieties of CSOS. Several limiting factors such as medium reaction, time reaction, pH, and the molar ratio of reagent and cross-linker reaction can be altered, translating to the modified grafting behavior and degree of substitution of CSOS into oxidized NCC. Electrostatic interactions were performed to achieve 21.5% of binding efficiency loading and 14% *w*/*w* of procaine hydrochloride (PrHy) loading. The rapid release profile observed in this study is suitable for local drug delivery by the oral system [159].

**Figure 7:**
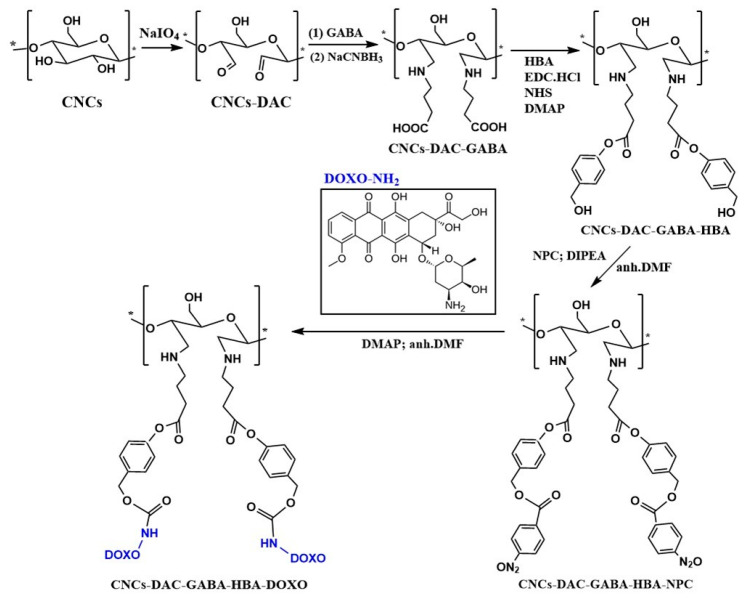
Illustrative representation of conjugated doxorubicin onto NCCs through chemical bonding (this picture is re-drawn from Tortorella et al. [160]. Copyright © Springer Fachmedien Wiesbaden GmbH). Abbreviations: NCCs = cellulose nanocrystals, DAC = cellulose dialdehyde, GABA = c-amino butyric acid, HBA = 4-hydroxy benzyl alcohol, EDC HCl = 1-ethyl-3-(3-dimethylaminopropyl) carbodiimide hydrochloride, NHS = *N*-hydroxy succinimide, DMAP = 4-dimethylamino pyridine, NPC = 4-nitrophenyl chloroformate, DIPEA = *N*,*N*-diisopropyl-*N*-ethylamine, anh. DMF = anhydrous dimethylformamide, DOXONH2 = doxorubicin, * is repetitive monomer molecules.

Direct covalent drug attachment towards the NC crystal backbone via a novel spacer arm through amine-mediated couplings is another potential strategy [160]. Tortorella et al. [160] modified NCC via periodate-oxidation-generated NCC-DAC (dialdehyde cellulose) and inserted them into molecules of g-aminobutyric acid (GABA) via the Schiff base condensation reaction. Subsequently, the nucleophilic substitution of 4-hydroxy benzyl alcohol (HBA) occurred and was followed by an acylation reaction with 4-nitrophenylchloroformiat that exerted a carbonate group for nucleophilic substitution of amino contained doxorubicin as model drug nucleophilic. Carbamate linkage adjacent to the linker presents highly stable conditions in an aqueous environment with harsh conditions, either basic or acidic. The drug release of active drugs was achieved only by hydrolysis in cells utilizing suitable enzymes to cleave a carbamate linkage (Figure 7).

### 5.4. Polymer Grafting Modified Nanocellulose

Polymer-grafted NC has been introduced as the sought-after functionalization strategy to refine the drug delivery performance. Different techniques have been developed to introduce functional groups onto NC covalently, i.e., (i) Thiolene reaction; (ii) Oxime reaction; (iii) Michael addition; and (iv) imine and hydrazone synthesis. These reactions have been well-developed for polymer functionalization for drug delivery systems.

Integrating polymer onto the NC surface can be performed by the ‘grafting onto’ or ‘grafting from’ strategy. The ‘grafting onto’ technique requires pre-synthesized polymer attachment that can bear the reactive end groups onto either modified or non-modified hydroxyl groups of the NC surface. The adherence of polymer onto the NC surface’s specific moieties can be performed via physical or chemical attachment. The ‘grafting onto’ approach offers the possibility of characterizing polymer before grafting and modulating the resultant carriers’ characteristics.

Strong electrostatic interaction can be used to initiate the polymer grafting onto NC. There is a possibility of incorporating polydopamine (PDA) into the NCC surface to fortify the PDA material and develop NC’s colloidal stability. The presence of functional groups in PDA, such as amine, imine, and catechol groups, can serve as the anchors for NC and the drug through the Van der Waals interaction, the *π–π* interaction, and hydrogel bonding [161].

**Figure 8:**
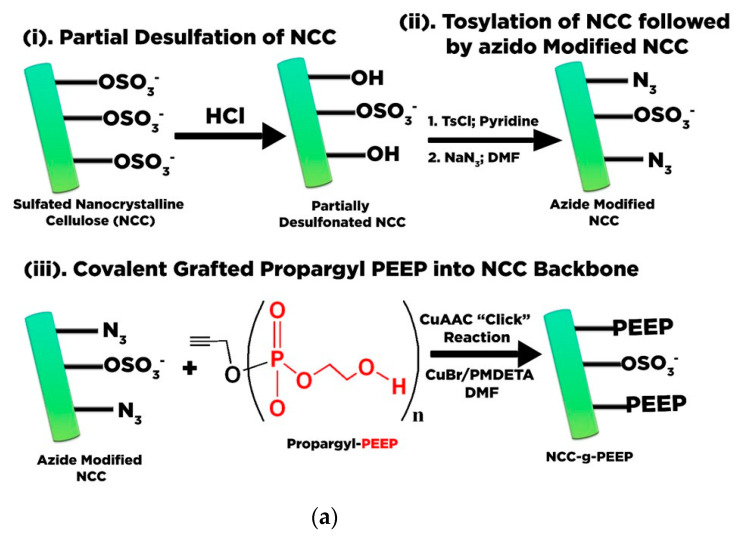

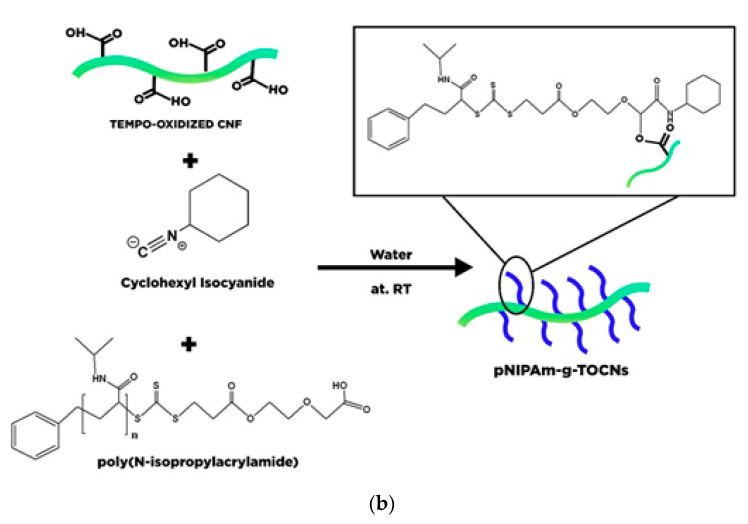
Schematic representation of the polymer grafting technique (**a**). CuAAC “click” reaction for NCC-gPEEP synthesis (this picture is redrawn from Wang et al. [162], Copyright 2010 Royal Society of Chemistry); (**b**) polymer-grafted cellulose fibrils (pNIPAm-g-TOCNs) via Passerini one-pot reaction (this figure is redrawn from Khine et al. [164]. Copyright © 2018 American Chemical Society).

Wang et al. [162] assembled poly(ethyl ethylene phosphate) (PEEP) that bears propargyl functionality onto azide modified nanocrystalline cellulose (NCC) Cu via Cu(I)-catalyzed azide-alkyne cycloaddition (CuAAC) “click” chemistry. In parallel, azide-modified NCC was constructed by two steps, i.e., (i) partial desulfation treatment of NCC followed by tosylation (NCC-Cl); and (ii) conversion of NCC-Cl into azido-NCC through nucleophilic substitution using sodium azide. Propargyl-PEEP was grafted onto azide modified NCC (NCC-g-PEEP) (Figure 8a). The as-synthesized suspension with negative charge can be utilized for doxorubicin (DOX) confinement through electrostatic interaction, exhibiting pH-responsive delivery in the tumor cell environment [162].

Kumar et al. [163] explored Diels-alder “click” chemistry by attachment of the metronidazole drug onto the CNFs. Initially, the TEMPO-oxidized CNFs underwent amidation with furfuryl amine. Subsequently, esterification occurred between metronidazole as a drug model and maleimide-hexanoic acid to introduce the ester function between the drug and the maleimide ring. Finally, the Diels–Alder reaction occurs between the furan functionalized CNF-t (CNF-fur) and metronidazole containing maleimide. Thus, the novel system of carrier provides the ester function on the linking chain for innovative drug carrier formulation, which induces the release in the presence of esterases enzyme [163].

A versatile grafting strategy for numerous functional groups is the Passerini reaction. This reaction is a multicomponent reaction (MCR) that comprises three substances, i.e., a carboxylic acid, an isocyanide, and aldehyde/a ketone, in one pot of reaction. For example, Khine et al. [164] modified poly(*N*-isopropylacrylamide) pNIPAm carrying aldehyde end groups via the Reversible Addition−Fragmentation Chain Transfer (RAFT) polymerization technique. Subsequently, the polymer with aldehyde functionality was further chemically grafted into TEMPO-oxidized CNFs. As a result, these materials exhibit thermal responsiveness, which is promising for use in stimuli-responsive carriers (Figure 8b) [164].

Another way of modifying NC with polymer in an aqueous solution is the NICAL reaction. For example, Khine et al. [132] demonstrated photo-induced “click” chemistry for (TEMPO)-oxidized CNF bearing carboxylic acid moieties (TOCNs) modified with the nitrile imine-mediated tetrazole under ultraviolet (UV) irradiation. The presence of fluorescence characteristics allowed for direct monitoring of NC throughout the cancer cells’ incubation. In addition, doxorubicin as a drug model can be attached via electrostatic interaction to introduce excess negative charge onto carboxyl groups in the polymeric-grafted NC [132].

Undesirable reduction in surface grafting density is nonetheless observed as the major limitation. The steric barrier can hinder the optimum grafting throughout the reaction because the layer of attached polymer covered the available active sites. Therefore, an alternative strategy has been proposed, called ‘grafting from’. Using this method, the polymer chains can be grown in situ on the surface hydroxyl groups of NC via ring-opening polymerization (ROP) with the presence of stannous octoate (Sn(Oct)_2_) as an ROP agent. Another approach is atom transfer radical polymerization (ATRP) with 2-bromoisobutyrylbromide (BIBB) as the ATRP agent. These standard approaches for drug delivery have been well-reviewed elsewhere [132].

### 5.5. Surfactant Modified Nanocellulose

The adsorption of surfactants represents a promising alternative for the chemical modification of NC. Surfactants are classified into cationic, anionic, zwitterionic, and non-ionic. The distinct properties of the surfactant manifest through its micelle formulation in the aqueous solution, which is highly beneficial in the drug delivery system. The lack of a strong covalent bond is considered the significant drawback towards enabling molecule release. Therefore, it is necessary to study several factors affecting the interaction of surfactant and NC and their impact on drug uptake and release. Tardy et al. [165] reviewed several factors that influence the affinity of NC and the surfactant. This study provides some additional information on the affinity of NC and the surfactant on the drug delivery system.

The opposite charge between the NCC surface and CTAB drove the electrostatic interaction and physical adsorption for the NCC surface modified with the surfactant. NCC’s negative charge creates a non-covalent interaction towards the cationic charge of CTAB, resulting in a strong electrostatic interaction. Zainuddin et al. [166] pointed out several factors that mainly involve the interaction between NCC and the surfactant, i.e., pH and ionic strength, the CTAB concentration, and the ratio of CTAB to NCC. They highlighted that the CTAB concentration and mass ratio of NCC: CTAB affects the interaction of surfactant-modified NCC with curcumin as a hydrophobic drug model. Increasing the CTAB concentration intensifies the hydrophobic character of the carrier, which is intensely coordinated with curcumin. However, at a high concentration of CTAB, the amount of curcumin attached tends to decrease [166]. 

**Figure 9:**
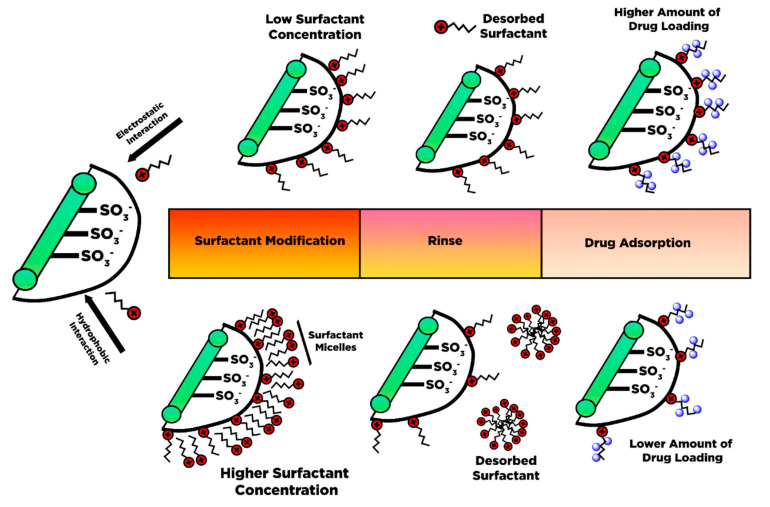
Schematic representation of the surfactant and nanocrystalline cellulose mechanism and its effect on drug adsorption (this figure is re-drawn from Bundjaja et al. [26]. Copyright © 2020 Elsevier B.V.).

Low surfactant concentration favors the electrostatic interaction between the monomer CTAB head with the negative charge of NCC surface, giving hydrophobic properties. While the CTAB concentration increases progressively, the adsorbed monomer of the surfactant tends to restructure and initiate surfactant cluster formation induced by hydrophobic coordination between surfactant alkyl chains. The CTAB cluster molecules can be absorbed through the NCC surface by hydrophobic interaction. However, the hydrophobic coordination of the surfactant and NCC manifested as a weak electrostatic interaction, which easily releases CTAB from NCC surfaces through the washing. Moreover, an excessive amount of CTAB concentration over the boundary of the surfactant critical micelles concentration (0.93 mM CTAB) might provoke the surfactant micelles formation on the NCC surface, which degrades the hydrophobic characters. Only ionic interaction between the cationic head of CTAB and anionic sulfate ester groups remains unaffected, which acts as available active sites for hydrophobic drug loading (Figure 9).

Raghav and Sharma [167] reported the coordination of the hydrophobic tail of CTAB in phosphate NCC. They also observed that the surfactant types (CTAB and TBAB) influence the capability of modified NCC to bind and release the drug. By observing the structure configuration, stearic near the central nitrogen in TBAB-NCC causes the insufficiency of drug binding, which exacerbates the coordination and controlled release of the carrier [168].

Putro et al. [25] modified the NCC with various types of surfactants such as cationic (CTAB), anionic (sodium dodecyl sulfate), and non-ionic surfactant (Tween 20). Different types of surfactants exhibit distinct interactions towards NCC, which influenced the electronegativity of modified NCC itself and the drug adsorption–desorption behavior. The presence of salt in the system had a significant influence on the uptake of paclitaxel. Different behavior of surfactants due to the salt effect significantly influences the interaction of NCC and drugs. They concluded that (1) electrostatic and Van der Waals interactions are the primary mechanism of paclitaxel adsorption towards surfactant-modified NCC, which can be enhanced through salt addition; and (2) pH played a significant role in the drug adsorption and release of paclitaxel by altering the surface charge of surfactant-modified NCC and the electrostatic interaction of hydroxyl ions and paclitaxel in solution.

Surfactants have also been widely used to modify cellulose nanofiber for poorly soluble drug adsorption performance. The surfactant attachment on the CNF surface is vital to overcome various limitations of CNF’s chemical and physical characteristics during modification for drug conveyance systems. The physical interaction of the surfactant and CNF may overcome the aggregation tendency of CNF in an organic solvent, thus increasing the solvent’s ability to assist CNF modification and adsorption of hydrophobic drugs. The presence of the surfactant strengthens the cationic and hydrophobic characters of CNF. A carrier’s physical and chemical characteristics can be refined by adding a surfactant (CNF film and foams-based CNF).

The synthetic surfactant can induce membrane cell lysis based on biocompatibility, which is considered a toxic material for cells. Therefore, the naturally available surfactants have been considered to replace synthetic surfactants given their low toxicity. For instance, Bundjaja et al. [26] utilized natural surfactant (rarasaponin) extracted from *Sapindus rarak* DC fruits to modify nanocellulose via hydrophobic interaction. The results of their study indicated that the *rarasaponin*-modified NCC exhibits a lower adsorption capability of tetracycline relative to synthetic surfactant-modified NCC (CTAB, Tween20, and SDS). The utilization of natural surfactants for the modification of nanocellulose materials remains a challenge. Other bioactive compounds that were attached to the surface of NCC may cause limited interaction for tetracycline molecules.

### 5.6. Polyelectrolytes-Based Nanocellulose

Polyelectrolytes are charged polymers in which their repeating units contain the electrolyte group. In polar solutions such as water, these polymers dissociate into cations or anions. The most common approach to make a functional polyelectrolyte carrier is to create a multilayer carrier through electrostatically assembling layer-by-layer (LbL) the nanocellulose (either negative or positive surface) with an oppositely charged polyelectrolyte. Currently, the development of drug delivery carriers through LbL assembly has drawn considerable attention due to their unique properties. Various physical interactions such as hydrogen bonding, hydrophobic interaction, and Van der Waals interaction are present in functional polyelectrolyte carriers. Those interactions act as the driving force in drug binding and maintain the stability of the multilayer [169]. LbL hybridization assembly of nanocellulose with other organic and inorganic materials usually instigates an outstanding performance improvement for the entire LbL system to stimuli-responsive and localized drug delivery. Early development of the LbL approaches was demonstrated on the flat substrates and is currently extended to spherical particles.

Coating LbL film on a spherical sacrificial template becomes another layer-by-layer assembly approach for hollow polyelectrolytes capsule formation to encapsulate and release the drug. Melamine formaldehyde (MF) is a popular template for microcapsules preparation via LbL assembly due to narrow-sized distribution and optimized dissolution conditions [170]. The physicochemical characteristics of templates such as size, shape, porosity, colloidal stability, and template solubility modulate the characteristic of as-synthesized hollow capsules. For instance, the capsule size can be adjusted depending on the size of the template, which is common in the range of 150 nm to a few micrometers [171]. Nanocellulose has been used to construct the interior of multilayer thin film and hollow microcapsules for various types of therapeutic molecules loading such as DNA, RNA, protein, and drugs.

Several aspects should be considered to assemble suitable polyelectrolyte complexes through the LbL system, i.e., charge stoichiometry, charge density, molecular weight, polyelectrolytes concentration, pH, ionic strength, order of addition, mixing ratio, and mode of mixing the polyelectrolyte solution. These factors greatly influence the drug carrier thickness, the surface charge, and the morphological structure, such as the size, shape, and porous structure of the drug delivery system. Reviews on some crucial aspects that influence the stability of polyelectrolyte complexes for drug delivery systems are available elsewhere [172].

Mohanta et al. [173] produced an NCC multilayer thin film with counterionic polyelectrolytes (chitosan) on a quartz crystal microbalance (QCM) plate through LbL growth assembly. They also developed hollow microcapsules using MF as a template. By varying the concentration of the polyelectrolyte (either NCC and chitosan) and the number of depositions, a homogeneous multilayer thickness with a porous structure can be obtained. The thin film and microcapsule were utilized as carriers for hydrophilic drugs (doxorubicin) and hydrophobic drugs (curcumin). The protonation of amine groups in acidic conditions becomes the driving force for doxorubicin release, while the concentration difference between the medium and carrier is considered the primary factor affecting curcumin release. The stimulus-responsive pH in LbL system-based nanocellulose may apply to local drug transport and tumor therapy [173].

Other types of layer-by-layer assembly approaches were also used to construct PEC-based nanocellulose by incorporating various types of polyelectrolytes. For instance, Li et al. [174] proposed the buildup technique of LbL for opposite-charge building blocks (e.g., cellulose nanocrystal (NCC), polyethyleneimine (PEI), cis-aconityl-doxorubicin (CAD), and building blocks of folate (FA)). The highly negative charge of NCC serves as an anchor to carry the positive-charge PEI through electrostatic interaction as an intermediary layer. The coordination of NCC-PEI resulted in positive-charge material for electrostatic adsorption of the negative charge of FA and CAD to construct the outermost layer, which took place sequentially (denoted as FA/CAD@PEI@NCC). The presence of FA on the surface carrier increased the active targeting ability towards folate receptors in the tumor cell. Cis-aconityl amide linkage in doxorubicin (CAD) can specifically release DOX at the lysosomal pH due to the pH labile characteristic and hydrolysis cis-aconityl amide linkage by β-carboxylic acid under low pH. The integration of each layer can increase the uptake to 20 times larger than its counterpart due to the strong electrostatic charge. Besides the surface chemistry carrier, the carrier’s morphological structure also helps the carrier delivery reach tumor cells [174]. Another potential form of polyelectrolytes, including hydrogel, aerogel, lightweight porous materials, and integrated inorganic–organic composites, are thoroughly discussed in the following sections.

## 6. Hydrogel Based Nanocellulose for Drug Delivery

Hydrogels are three-dimensional (3D) cross-linked polymeric networks that carry absorbed water and store a large quantity of water in the swelling state. The hydrogels can be cross-linked through physical (non-covalent interaction), chemical (covalent coordination), or an integration of both physical and chemical cross-links [175]. Given its biocompatibility and stimulus-responsive swelling behavior, the hydrogel has gained attention for drug delivery application. As a drug carrier the physically cross-linked hydrogel is preferable to the chemically cross-linked hydrogel. The covalently cross-linked hydrogel generates a permanent structure that limits the swelling ability, and therefore, most chemically cross-linked hydrogels are used as implantables. Furthermore, the incorporation of the drug via adsorption towards chemically cross-linked hydrogel restrains the loading efficacy. Although the cross-linked reaction may perform drug conjugation on the hydrogel, it sacrifices the chemical integrity of the drugs. Therefore, it is more desirable to construct a hydrogel delivery system where simultaneous gel formation and drug adsorption can occur in an aqueous environment without covalent cross-linking.

Due to the presence of sol-gel transition characteristics (such as swelling behavior, mechanical strength, and network structure), which are affected by the external stimulus such as pH, thermal, light wavelength, ultrasonic waves, pressure, magnetic field, and electrical field; the smart hydrogel-based nanocellulose has been well-developed for various drug delivery formulation. Diverse types of polyelectrolytes can modify the substantial charge of nanocellulose (either positive and negative) to form a variety of intelligent hydrogels such as injectable hydrogel [161], stimuli-responsive hydrogel [176], double-membrane hydrogel [177], supramolecular hydrogel [178], microsphere hydrogels, bacteria cellulose hydrogel [179], shape memory-based bacteria cellulose [180], and aerogel/cryogel [174]. All those hydrogels have desirable physical and chemical characteristics to be adapted to various drug delivery systems. Liu et al. [161] reviewed the current development of nanocellulose-based hydrogel and its modification for drug delivery systems. However, double-membrane hydrogel and supramolecular hydrogel are excluded from their review [161].

**Figure 10:**
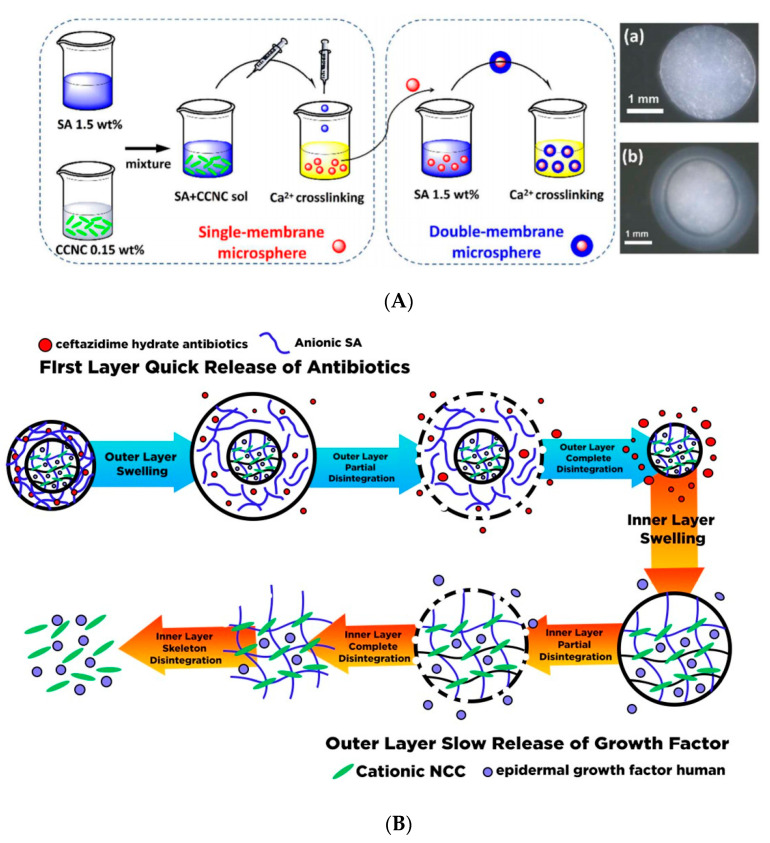
(**A**) The route fabrication of single membrane and double-membrane microsphere hydrogel with its optical microscope of single membrane SA/CNCC microsphere hydrogel and SA/CNCC double-membrane microsphere hydrogel (this figure is reprinted with permission from ref. [177]. Copyright © 2016 American Chemical Society); (**B**) schematic illustration of dual drug release mechanism from a double-layer membrane hydrogel constructed from Cationic NCC and Alginate (this figure is redrawn from Lin et al. [177]. Copyright © 2016 American Chemical Society).

Different types of hydrogels have diverse morphological structures, network coordination, and functional groups, affecting the drug’s diffusional path during adsorption and release. Double-membrane hydrogel was developed by Lin et al. [177], consisting of an external membrane composed of alginate and consolidation of cationic NCC (CNCC). Two different drugs were introduced on different layers of the membrane with contrasting types of release behavior. The outer hydrogel releases the drug rapidly, while sustained drug release occurs in the inner membrane hydrogel. This phenomenon occurred due to the ‘nano-obstruction effect’ and ‘nano-locking effect’ induced by CNCC components in the hydrogels. The ‘nano-obstruction effect’ offers sustained drug release throughout fragmentary disintegration, and the ‘Nano-locking effect’ is responsible for restricting the burst of drug release through progressive hydrogel disintegration (Figure 10). The different compositions and properties of external and internal hydrogels affect the drug’s behavior and diffusional path [177].

Supramolecular hydrogels have been characterized as the 3-D solid network hydrogel organized by non-covalent interactions such as hydrogen linkage, hydrophobic coordination, and cation-π and π-π interactions. In contrast to the chemically cross-linked hydrogels, gel morphology is equilibrated through covalent coordination; the supramolecular hydrogel morphology is stabilized by a non-covalent interaction. Supramolecular hydrogel has been synthesized through extensive, diverse supramolecular configurations, including host–guest complexation, biomimetic interaction, hydrogen bonding, stereo-complex formation, and ionic and metal-ligand. Hydrogel-based supramolecular self-assembly through host–guest complexation is the most widely explored method for supramolecular hydrogel formation. Specifically, supramolecular hydrogels constructed by host–guest inclusion between polymer and cyclodextrin demonstrated the thixotropic reversibility, which is advantageous for syringe drug delivery.

**Figure 11:**
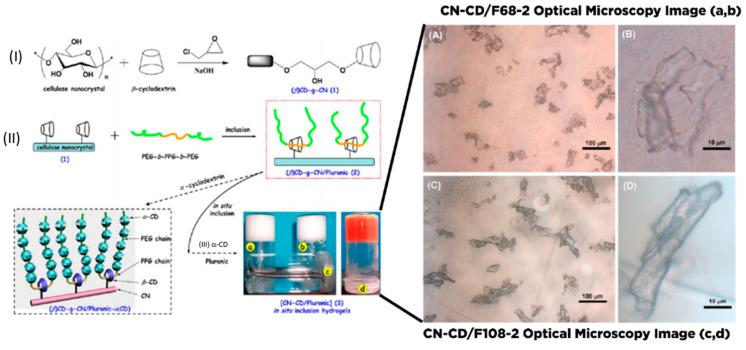
Construction pathway of (**I**) cellulose nanocrystal (β)CD-g-CN grafted β-cyclodextrin; (**II**) complex inclusion between Pluronic polymers and (β)CD-g-CN; (**III**) supramolecular hydrogels comprising an in situ inclusion between (β)CD-g-CN/Pluronic and α-CD (**a**) hydrogel CN-CD/F68-2 and its and its morphological evidence, (**b**) hydrogel CN-CD/F108-2 and its morphological evidence, (**c**) water, (**d**) drug-loaded hydrogel CN-CD/F108-2-Dox. This figure is reprinted with permission from [178]. Copyright © 2013 American Chemical Society.

**Figure 12:**
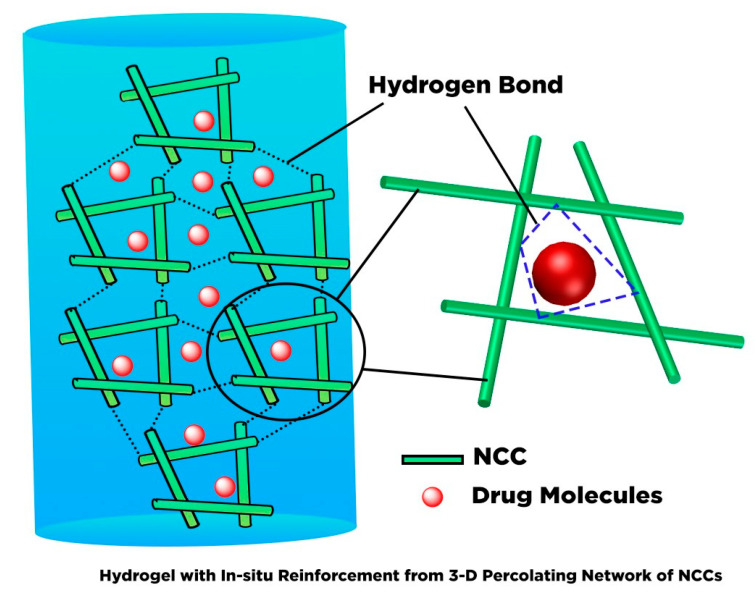
Schematic illustration of possible locking effect of the drug via host–guest inclusion in supramolecular hydrogel constructed from cyclodextrin and chemically modified nanocrystalline cellulose (this picture is redrawn from [178]. Copyright © 2013 American Chemical Society.)

Lin and Dufresne et al. [178] produced supramolecular hydrogel DDS by self-assembly of a covalently grafted α-cyclodextrin (α-CD) NCC surface with epichlorohydrin as a coupling agent through a one-step process. Furthermore, pluronic composed of triblock copolymers with different molecular weights (Pluronic F68 or F108), both bearing hydrophobic poly-(propylene glycol) (PPG) and hydrophilic poly(ethylene glycol) (PEG) segments (PEG-b-PPG-b-PEG), were immobilized on NCCs via the inclusion interaction between the hydrophobic segment of polymer and cyclodextrin (Figure 11). The supramolecular hydrogel-based NCC was utilized as a drug carrier for anti-cancer in vitro release of doxorubicin, which exhibited sustained drug release behavior (6.5 days). The kinetic release mechanism follows the ‘obstruction’ and ‘locking’ effects. They found that supramolecular hydrogels, upon being modified with NCC, induce a physical obstruction effect. Moreover, the adequate loading of NCC gave strong interaction (e.g., hydrogen bonding) inside supramolecular hydrogels and enabled the polymers to associate in the tridimensional percolating network, which provides a “locking effect” to delay the diffusion of doxorubicin molecules (Figure 12). The sustained release depends on the a-CD content, the chain length of the pluronic polymer, and the amount of NCC loaded in supramolecular hydrogels [178].

**Figure 13:**
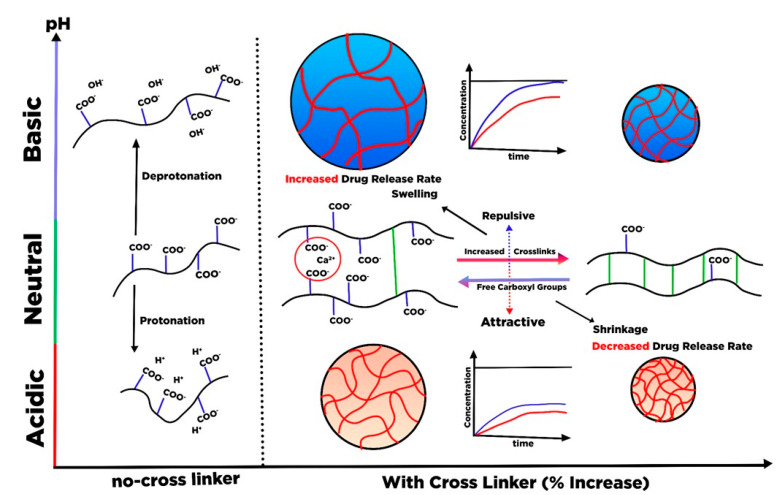
Schematic illustration of the swelling mechanism of hydrogel fabricated from TEMPO-mediated CNFs and alginate towards drug release (this figure is redrawn from [181]. Copyright © 2020 Elsevier B.V.).

Kopac et al. [181] pointed out that the main parameter for controlling the drug delivery rate in an anionic hydrogel-based nanocellulose is the average pore size (mesh size), controlled by selecting cross-linked and biopolymer concentration along with the adjustment of pH and temperature. The changes in the ionic strength and hydrogen bonding of functional groups in the internal hydrogel structure are responsible for altering the polymeric hydrogel network, which affected the average pore size of hydrogel (Figure 13). Due to the smaller hydrodynamic size of the drug relative to the mesh size, the drug can rapidly diffuse through the hydrogel network and vice versa without a steric barrier. However, both drugs can have a similar drug release rate by modulating the mesh size through cross-linking density and biopolymer ratio variation [181].

## 7. Lightweight Porous Based Nanocellulose for Drug Delivery

Lightweight porous materials have been classified as a 3-D solid class of material with several features such as high specific surface area, very low density (<50%), and diverse pore structure with various pore sizes ranging from nanometer to micron. Sponge, foam, and aerogels are the three major categories of lightweight porous materials. The sponge is constructed by gas dispersion in the solid matrix, commonly present as an open cell structure of low density porous elastic polymer. The sponge has a macroporous structure full of gaps and channels, permitting easy access to water or molecules flow [182]. Similarly, foam can be made through the steady gas dispersion into a hydrogel or solid matrix and even liquid. Foam is commonly characterized as having a bubble diameter (pore diameter) greater than 50 nm [183]. Aerogel is a three-dimensional (3D) porous material constructed by self-assembly of the colloidal component or polymeric chains, creating nano-porous networks that can be filled up with a gaseous dispersion medium. Aerogel is prepared through the wet-gel drying process by removing the liquid component in the hydrogel, which is replaced by a gas constituent while still preserving the gel network [184]. The specific surface area of aerogel can reach up to 1000 m^2^ g^−1^ with a porosity range between 80 and 99.8%. On the other hand, other aerogels, namely xerogel and cryogel, have been prepared by evaporation and freeze-drying. Detailed preparation of light-weight porous material-based nanocellulose has been reviewed elsewhere [185].

For the drug delivery field, carrier morphology, especially the porosity structure, controls the drug adsorption and release since the drug will pass through the internal pore to be retained inside and release outside regardless of the chemistry interaction. Sun et al. [186] underlined that the critical factor in controlling and modulating the pore structure of ultralightweight porous materials is selecting a drying method [186]. Initially, freeze-drying, supercritical drying, and evaporation drying have been utilized in fabricating ultralightweight porous materials. Evaporation drying has emerged as a conventional technique of synthesizing nanocellulose-based porous materials. However, there are several major drawbacks, such as internal network structure collapse due to the capillary forces of the solid matrix and the difficulty to prevent the shrinkage. Therefore, freeze-drying and supercritical drying have been used as drying methods to overcome these drawbacks. Freeze drying can retain the porous structure through the sublimation of liquid into gas. It is also possible to cross the solid–gas interface bypassing the liquid critical point through adjusting the temperature and pressure (supercritical drying). Both methods effectively retain the pore structure and refine the porosity and specific surface area of nanocellulose-based porous materials.

Aerogel-, xerogel-, and cryogel-based nanocelluloses are promising materials as the vehicle for a drug delivery system. Before the drying process, physical and chemical cross-linking are vital in controlling the 3D network formation and porous material performance. Physical cross-linking is commonly established by weaker interactions such as Van der Waals, hydrogen bonding, and electrostatic interaction. In contrast, covalent cross-linking can create a 3-D robust mechanical framework through the action of covalent coordination and polymerization. Chemical cross-linking exhibits better mechanical stiffness and structural stability compared to physical cross-linking.

Muller et al. [180] synthesized water-responsive xerogel to retain its original shape by submerging it in water through moisture utilization as the stimulus. The post-modification of BNC with the different supplementary hydrophilic substances was performed to achieve the re-swelling behavior. Rapid re-swelling behavior can be acquired by supplementary magnesium chloride, glucose, sucrose, and sorbitol with up to 88% maximum rehydration. Their findings of re-swelling modified BNC showed the possibility of developing a carrier with controlled release properties for hydrophilic drug model azorubine in the drug delivery system.

Li et al. [174] synthesized two types of nanocellulose/gelatin composite cryogels through hydrogen bonding and chemical cross-linking with dialdehyde starch (DAS) for controlled drug delivery of 5-fluorouracil (5-FU). DAS subsequently reacted with both gelatin and CNF to form the chemically cross-linked network. The reaction of aldehyde groups with the hydroxyl groups of CNFs led to the formation of hemiacetal/acetal types of structures. Furthermore, the aldehyde group presence effectively integrates with the ε-amino groups of gelatin to generate a Schiff base coordination. They found that the chemical cross-linking of Schiff bases and hemiacetal/acetate is crucial to regulate the structural porosity of cryogel composite. Since the porosity and cross-linking degree mainly control drug loading, selecting the chemical cross-linking method is crucial.

**Figure 14:**
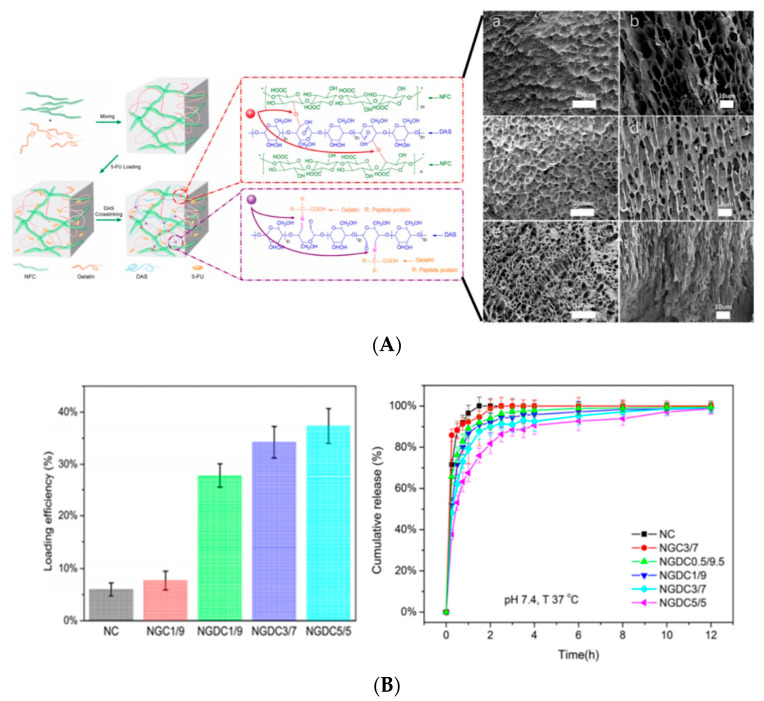
(**A**) Synthesis pathway and morphological structure of different ratio of NFC/Gelatin R_NFC/Gelatin_: (**a**,**b**) NGDC1/9; (**c**,**d**) NGDC3/7; (**e**,**f**) NGDC5/5. Surface (**a**–**c**); cross-section (**b**,**d**,**f**); (**B**) the influence of morphological structure of NFC/Gelatin Cryogel towards drug loading (left side) and release efficiency (right side) (This figure is reprinted with permission from [174]. Copyright © 2019 American Chemical Society).

Moreover, the presence of gelatin hydration capability and reversible hydrolysis characteristic of hemiacetal/acetate, along with its morphological structure, is also responsible for achieving controllable and sustained release of 5-FU in a simulated intestinal environment. In addition, the cross-linking degree and the porosity can be tuned by the composition and ratio of CNF, gelatin, and dialdehyde starch. The addition of CNF increases the drug loading and the cross-linking degree [174]. Figure 14A shows that the improvement of surface roughness and cross-section morphology reduces the pore size of cryogel, leading to an increase in the cryogel resistance against ice crystal growth during freeze-drying, resulting in the smaller pore size, higher specific surface area, and lower density. The smaller pore size leads to better drug loading and releases efficiency since the smaller pore structure limits the drug looseness (Figure 14B).

Zhao et al. [187] prepared polyethyleneimine (PEI) grafted to amine-modified CNF and cross-linked using glutaraldehyde to form an aerogel (CNFs-PEI). The success of the aerogel formation depends on the polymerization of methyl methacrylate (MMA) on the surface of CNFs, which induced the formation of the network between PEI and CNF. Polyethyleneimine (PEI) carries some primary and secondary amine functional groups, which increase the loading of sodium salicylate (NaSA) to 20 times higher than its counterpart (CNFs-based aerogel). The sustained and controlled release was achieved by the CNFs-PEI aerogel, which is highly responsive to pH because of the protonation and deprotonation of amine groups in PEI [187].

Chemically cross-linked PEI with TEMPO-mediated BC CNF (abbreviated as PEI-BC) for aspirin, gentamicin, and bovine serum albumin (BSA) carrier has been studied by Chen et al. [156]. The PEI cross-linking induced the morphological changes of BC by increasing the density of interconnected structures and thickening the pore walls, which provide the CNF interpenetrated network with improved mechanical strength [156]. Liang et al. [188] proposed a well-balanced dual responsive polymer (temperature and pH) by modifying branched PEI with *N*-isopropyl acrylamide (NIPAM), which was further grafted onto CNF through the condensation reaction (abbreviated as CNF-PEI-NIPAM). Remarkably, the pH and temperature of the carrier can alter the hydrophobic and hydrophilic characteristics of CNF-PEI-NIPAM [188].

CNF has been combined with the non-edible surfactant to make air bubble confinement by the Pickering technique, generating stable air bubbles encapsulated in wet-stable foams. Using the unique drying technique, the dry-foams with closed holes (cellular solid material-CSM) were made. Although the three-dimensional closed-hole structure presents a fascinating drug delivery system for the prolonged release of the drug because of confined stable air in the internal foam’s structure, such structure may induce an elongated diffusional path of medicine to modify the characteristic drug release. CNFs foam as a drug carrier with the positive buoyancy characteristic was synthesized by Svagan et al. [189]. Positive buoyancy characteristics resulting from the presence of air are retained in the closed cells. These primary characteristics highlight the practicability of CNFs foam as a floating agent for gastro retentive drug delivery systems for site-specific drug release such as intestinal and stomach systems. 

**Figure 15:**
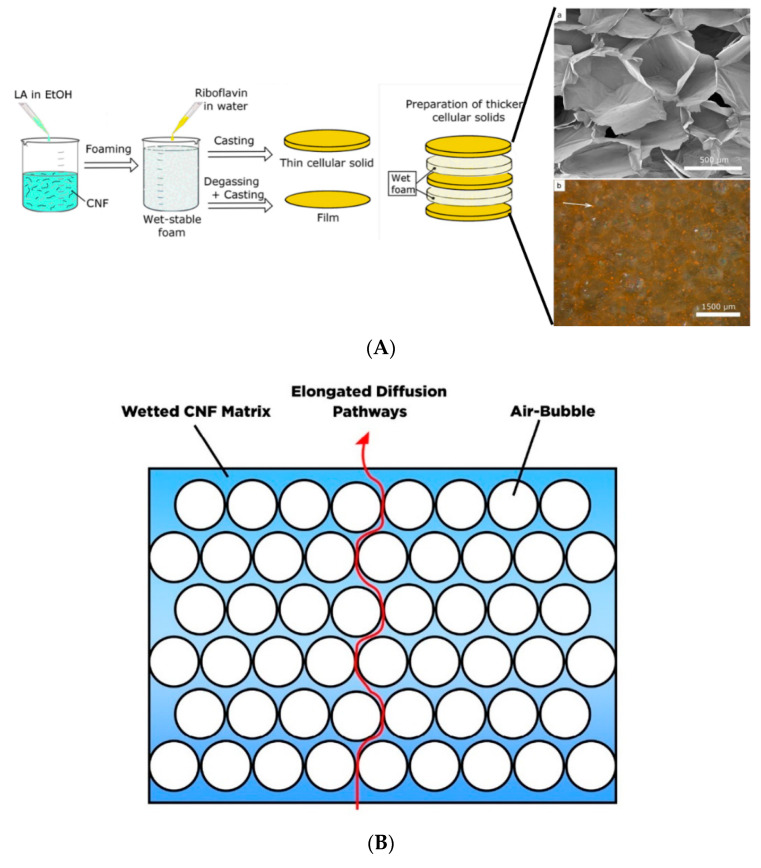
(**A**). Preparation route of CNF-based foams and its morphological, structural characteristic: (**a**): CNF-based foams cross-section morphological image; (**b**) cell structure image of CNF/LA loaded with riboflavin (the arrow points to riboflavin). (**B**) elongated diffusional pathways of the drug in foam-based CNFs (this figure is redrawn from Svagan et al. [189]. Copyright © 2016 Elsevier B.V.).

CNF foams were synthesized by combining the cationic suspension of CNF with the consumable surfactant (lauric acid sodium salt) as a foaming reagent. Subsequently, hydrophilic drug riboflavin was confined in the wet-stable CNFs foam structure and was further dried to acquire dry foam with a close hole structure with up to 50% drug loading (Figure 15A). The CNFs foam offers structural flexibility with different porosity and tortuosity, which can be modified in terms of shape and thickness and can be sliced into different pieces. An increase in the foam thickness leads to a decrease in the riboflavin release rate. In addition, the morphological foam structure showed a long and tortuous diffusion path, prolonging drug diffusion (Figure 15B). Therefore, the diffusion coefficient of the drug through the porous foam structure was lower than the diffusivity of the drug in the film structure [189].

The addition of surfactant is required to synthesize stable dry-foam-based cellulose nanofibers. Lobmann et al. [190] proposed an innovative way to synthesize stable foams by combining cationic CNF and hydrophobic drug indomethacin. Hydrophobic drugs provided a positive molecular interaction by partially covering the hydrophobic side of CNFs, which further changes the surface energy of CNFs. However, the indomethacin loading in the foams was limited to up to 21% of the loaded drug. An excessive amount of drug loading would destabilize and collapse the foam’s structure since a higher fraction of free indomethacin and solvent in the solution was present in the air–water interface, which limited the surface-modified CNF aggregation [190].

Svagan et al. [191] performed similar assembling of controlled-release CNFs foam with buoyance characteristics utilizing the poorly soluble drug furosemide as a foaming agent. They highlighted several factors such as the amount of drug loading, the foam piece dimension, and the solid-state of the incorporated drug that influenced the kinetic release of the drugs. Regarding the solid-state of the drug within the closed cell of foam, at 21% furosemide loading in foam, furosemide mainly exists in an amorphous state of furosemide salt, which leads to rapid release with the increase of the drug loading. In addition, the mass of incorporated drugs inside the foam structure can provide different foam dimensions, which alter the drug release kinetics. Bannow et al. [192] investigated the influence of processing parameters on the foaming characteristic and structure of nanofoam CNF/indomethacin. They found that the nanofoam density and the number of entrapped air bubbles depend on the pH, the mass of confined drugs, and the preparation route (pre- or post-adjustment of pH) [192].

**Figure 16:**
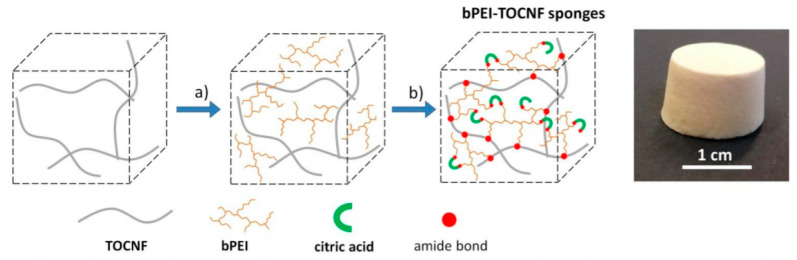
Preparation route of sponge-based TOCNFs via crosslinking of bPEI and TOCNFs with CA as a crosslinker; arrow (**a**) is cross-linking process, and arrow (**b**) is auxiliary carboxyl addition (this figure is reprinted with permission from ref. [193]. Copyright © 2017 Wiley-VCH Verlag GmbH & Co. KGaA, Weinheim.).

The development of sponge-based nanocellulose for the drug delivery system by adding citric acid (CA) as a co-cross-linker between branched polyethyleneimine (bPEI) and TOCNFs was conducted by Fiorati and coworkers [193]. CA was added as an auxiliary carboxyl moieties source to improve the cross-linking process to bPEI. They investigated the as-synthesized sponge capability as a drug vehicle for amoxicillin and ibuprofen. The confined drug in the sponge structure with non-contained citric acid moieties exhibited a higher drug release percentage than that with the cross-linker. The presence of citric acid progressively increased the ibuprofen adsorption, while no significant effect was observed for amoxicillin adsorption. The presence of citric acid provided an additional carboxylic group, which was actively involved in the particular interaction with the ibuprofen molecules. In addition, the existence of CA also refined the mechanical strength and chemical stability of the material through the occurrence of amide bond formation between the primary amines of bPEI and with carboxylic groups of TOCNFs and CA (Figure 16).

The progress of nanocellulose based sponges is still quite limited in the drug delivery field. Nevertheless, several types of sponge-based nanocellulose have been used in other biomedical applications. For instance, Xiao et al. [194] developed sponge-based CNFs via multiple cross-linking of CNFs by cellulose acetoacetate (CAA) and aminopropyl (triethoxy) silane (APTES), which further covalently bonded with surface-modified gentamicin through enamine coordination. The sponges’ composite exhibits outstanding antibacterial characteristics towards *S. aureus* and *E. coli*, allowing 99.9% sterilization capability [194].

## 8. Integrated Inorganic/Organic-Based Nanocellulose for Drug Delivery

Recently, magnetic nanocomposites in drug delivery, particularly in cancer therapy, have drawn considerable attention. The targeted delivery of antitumor agents towards cancerous tissues can be carried through the advanced hybrid material with stimuli or specific recognition characteristics to pass through the targeted sites selectively. Nanohybrids with the stimuli effect respond to the external stimulus (e.g., pH, temperature, magnetic, and ultrasound) and further alter their physiological characteristic to release the therapeutic agent with a specific concentration towards the affected tissues. Therefore, the treatment system and the drug specificity can be improved, contributing to lessening systemic toxicity. Nonetheless, the drug carrier biocompatibility, immunogenicity, toxicity, responsiveness to magnetic gradients, and proper drug transportability still need much improvement.

**Figure 17:**
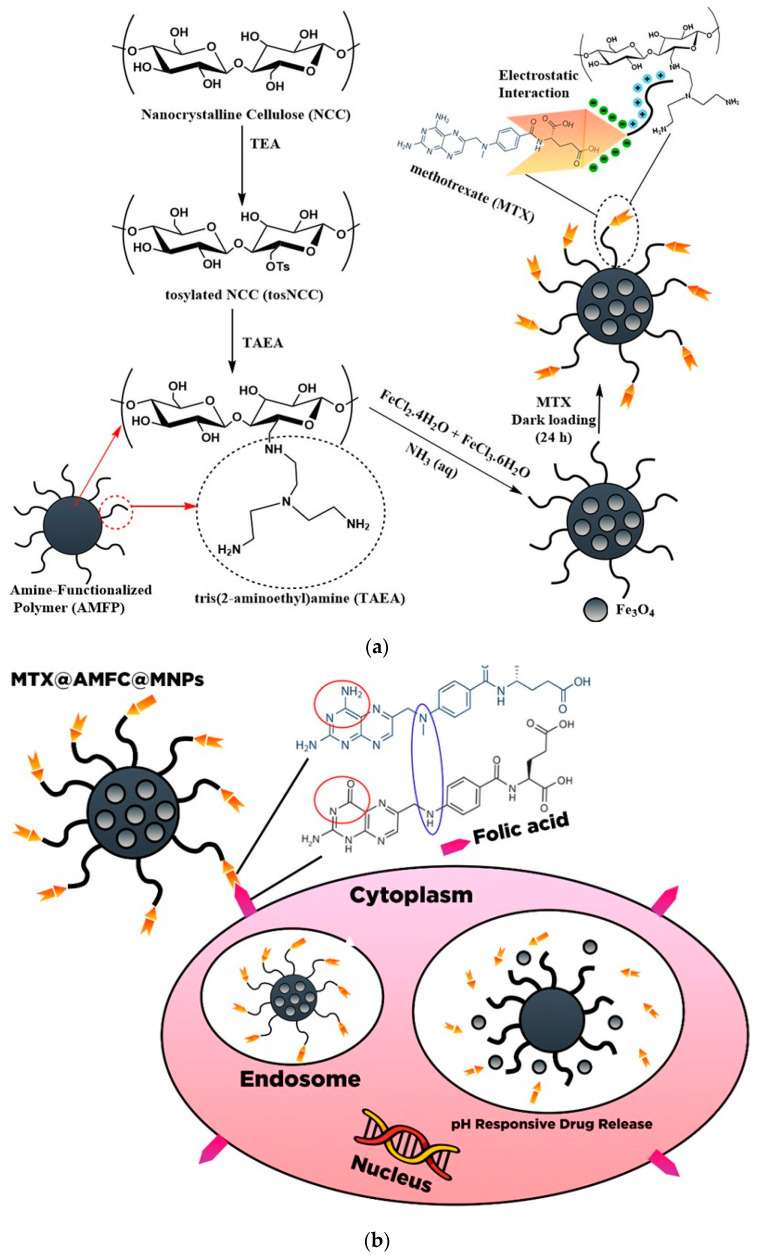
(**a**) Magnetic NCC-based nanocarrier with pH-responsive capability construction. The nanocellulose was undergoing tosylation, which reacted with tosyl chloride for tris(2-aminoethyl)amine (AMFC) functionalization, incorporating amino moieties for electrostatic interaction improvement, which connected into the methotrexate (MTX, anticancer drug) carboxyl groups (MTX@AMFC@MNPs); and (**b**) schematic illustration of pH-responsive and localization of cancer treatment that benefited from the structural similarity between folic acid and MTX, which assists the folate-receptor-mediated cell internalization (this figure is redrawn from [195]. Copyright 1987 Royal Society Of Chemistry).

NCC may also be utilized as a nanoparticle coating for colloidal stability improvement, biodegradability, biocompatibility, and chemical functionalization. Rahimi et al. [195] functionalized NCC with tris(2-aminoethyl)amine (AMFC) for Fe_3_O_4_ magnetic nanoparticles coating (AMFC-NPs). Initially, the nanocellulose underwent tosyl chloride treatment for tris(2-aminoethyl)amine functionalization (AMFC was chosen to be assigned to the amino moieties and cationic characteristics). The presence of amino groups in AMFC-NPs was linked to the methotrexate (MTX, an anticancer immunosuppressive drug) carboxyl groups. This method was employed to surpass the MTX limitation by keeping down the off-target side-impact towards healthy cells while optimizing the efficacy of anticancer drug delivery. The drug confinement efficacy reached 91.2% with 30.4% efficiency of drug loading in the AMFC-NPs. The MTX-AMFC-MNPs system exhibited pH responsivity in which, at an acidic condition (pH of 5.4), up to 79% of the drug was released, while over five days, it exhibited up to 29% drug release by the protonation behavior of MTX carboxylic groups. In addition, the nanoparticles containing MTX exhibit a higher uptake of cellular compared to the AMFC-MNPs, which were contributed to by the chemical similarity of MTX with folic acid (FA), which assists the internalization of receptor-mediated cellulose. This enhances the potential of MTX for cancer cell targeting (Figure 17) [195].

Recently, Supramaniam et al. [196] introduced magnetic characteristics towards nanocellulos-based hydrogels, which were further utilized for controlling drug delivery. The co-precipitation with Fe (II) and Fe (III) ions was incorporated into the NCCs, followed by the insertion of the magnetic characteristic, and subsequently, morphologically modified into beads with sodium alginate. It was observed that the magnetic nanocellulose existence refines the physical and mechanical characteristics of hydrogel beads and swelling degree improvement and limits the drug release due to the formation of physical entanglement inside the hydrogel. Jeddi and Mahkam [197] developed magnetic hydrogel beads composite-based carboxymethyl nanocellulose to deliver dexamethasone. The composite can control the dexamethasone delivery up to 12 h.

Carbon nanotubes have outstanding characteristics such as high thermal stability, homogenous pore arrangement, high specific area, and excellent electrical features. This advanced material has also been employed as a vehicle in the drug delivery system in recent years. The combination of this material with the NC material provides some advantages. The incorporation of nanocellulose in the composite increased biocompatibility and biodegradability while the CNTs provided good stability, magnetic and electromagnetic behavior, and high cellular uptake [198]. Although the cytotoxicity of material still became an issue, CNTs were widely exploited in drug delivery systems, particularly cancer therapy applications [199].

Integration of nanocellulose into graphene-based materials through the layer-by-layer assembly as a drug carrier was carried out by Anirudhan et al. [200]. Chemically modified GO was used as a template for the layer-by-layer assembly of aminated nano-dextran (AND) and carboxylic acid functionalized nanocellulose (NCCs) to form a MGO-AND/NCCs nanocomposite. Curcumin can be loaded into the carrier through π–π stacking and hydrogen bonding interactions due to the phenolic and aromatic rings of curcumin. Based on the release study, the acidic environment promotes COO- groups’ protonation and amino in aminated in nano-dextran to form NH_3_^+^. This phenomenon decreased the static interaction between MGO-AND/NCC, resulting in the electrostatic repulsion of each component, consequently provoking the drug release. In addition, a cytotoxicity assay on HCT116 cells exhibited high efficacy of curcumin-loaded MGO-AND/NCC.

The electrochemical activity of the carbon nanotube was utilized to modulate the drug release. The release of ibuprofen from a novel hybrid hydrogel composed of sodium alginate (SA), bacterial cellulose (BC), and multi-walled carbon nanotubes (MWCNTs) was studied by Shi et al. [176]. The release of ibuprofen can be provoked by electrostatic repulsion. Thereby, the on–off release mechanism can be attained by introducing electrochemical potential [176].

## 9. Conclusions

Modified and functionalized nanocelluloses with low toxicity and high biocompatibility render them promising materials as advanced drug carriers. Various hydroxyl groups on the surface of the nanocellulose serve as attachment sites of drugs through covalent and/or physical interactions. In addition, nanocellulose modification results in a different morphological structure for the carrier, which contributes to an increase in the diffusion pathway of the drug within the carrier. Therefore, surface chemistry is a crucial factor that should be considered in the design of nanocellulose as a drug carrier for effective drug delivery. High-purity nanocelluloses are also required to obtain drug carriers with the well-constructed framework, thus facilitating drug adsorption and release control. Considering all these factors, carrier-based nanocellulose is a promising candidate for developing novel sustained drug delivery systems.

## Figures and Tables

**Table 1:** Summary of the characteristics of various types of nanocelluloses.

	Types	Nanocrystalline Cellulose (NCC)	Cellulose Nanofibers (CNF)	Bacterial Cellulose
Parameter				
Common names		Cellulose whisker, cellulose nanowhisker, cellulose nanowire, and cellulose nanorod or spherical cellulose nanocrystals	Cellulose nanofibril, micro fibrillated cellulose, Nanofibrillar cellulose, Nanofibrillated cellulose, and cellulose microfibril	Microbial cellulose (MC), bacterial nanocellulose (BC), and bio-cellulose (BC)
Morphological structure		Needles like shape, elongated rod-like shape, and spindle shape	Smooth, extended, and flexible chain	Twisted ribbons like shape
Structure of Nanocellulose		Crystalline domains	amorphous and crystalline domains	Crystalline domains
Chain Length		≥500	500–15,000	4000–10,000
Crystallinity (%)		54–88	-	84–88
Other Impurities and contaminant		Possible to contain hemicellulose, lignin, and pectin	Possible to contain hemicellulose, lignin, and pectin	Contain no hemicellulose, lignin, and pectin
Size (Length and Diameter)		Diameter: 5–30 nm and Length: 100–500 nm	Diameter: 1–100 nm and Length: 500–2000 nm	Diameter 20–100 nm and several micrometric lengths
Process System		Top-down system	Top-down system	Bottom-up system
Tensile strength (Gpa)		7.5–7.7 [34]	13	0.2–0.3
Modulus Young (Gpa)		110–220 [45]	Approximately 15	18–20 [60]
Density (gr/cm^3^)		1.6 [61]	1.42	1.1
Characteristics		Homogenous nanorod form, exceptional aspect ratio (length to diameter), appreciable specific surface area (SSA), biocompatibility, liquid crystalline attribute, inferior breaking expansion, high young’s modulus, hydrophilicity, outstanding mechanical stiffness, tunable surface characteristic due to the reactive hydroxyl group and low density	Extended length with excellent aspect proportion (length to diameter), superlative surface area, hydrophilicity, biocompatibility and adjustable characteristic through surface modification afforded by high extensive of hydroxyl groups in CNF.	High crystallinity of nanocellulose (84–88%) and polymerization grade, high water uptake capacity (exceeding 100 times of its weight), remarkable surface area (high aspect proportion of fiber), outstanding tensile strength (young modulus 15–18 Gpa), and flexibility, foldability, moldability, mechanical stability, highly biocompatible material, non-cytotoxic, un-genotoxic and high porosity

**Table 2:** Summary of waste-based sources for nanocellulose production and its characteristic.

Waste Residue Sources	Nanocellulose Isolation Technique		Nanocellulose Characteristics	References
	Pretreatment	Treatment		
WASTE BASED FOREST RESIDUE				
Birch and Spruce sawdust	Hot water treatment and subsequent delignification; TEMPO oxidation	Mechanical defibrillation	CNF σ = 171,6 MPa; E = 6.4 Gpa;	[77]
Medium-density fiberboard	Soxhlet extraction (Ethanol and toluene), NaOH, and recurrent bleaching	Acid hydrolysis (H_2_SO_4_)	NCC L:164.7 nm; W: 6.7 nm; CrI (%): 71	[78]
Eucalyptus sawdust	Hot water treatment, alkaline delignification, O_2_ residual delignification, TEMPO-mediated Oxidation	High pressure homogenization	CNF D_avg_: 41.0 nm; SSA: 60 m^2^/g; Y (%) = 60	[79]
Pinecone biomass	Alkali treatment followed with acidification (NaClO_2_:CH_3_COOH)	Mechanical grinding.	CNF σ: 273 MPa; E: 17 GPa; CrI (%): 70%; D: 5–20 nm.	[80]
Logging residues	Alkaline and bleaching pretreatment	Acid hydrolysis (H_2_SO_4_)	NCC L/D > 10; CrI (%): 86–93; TS (°C): 208.4–211	[81]
Bamboo log chips	Pretreatment with glycerol; and screw extrusion	Mechanical refining/Milling treatment assisted by H_2_SO_4_ (0.15%) as a catalyst	CNF D: 20–80 nm; CrI (%): 52.7%; Y (%): 77.2	[82]
WASTE BASED ALGAE RESIDUE				
*Cladophorales*	-	TEMPO Oxidation;	CNF W: 80 nm; SSA: 77 m^2^/g CrI (%): 93%; D: 80 nm; Excellent mechanical and rheological characteristics	[83]
Red algae	-	Acid hydrolysis (H_2_SO_4_)	NCC L: 432 nm; W = 28.6 nm; L/D: 15.1; CrI (%): 69.5; Yield: 20.5%; TS (°C): 220 °C	[84]
*Green Seaweed* *Ulva lactuca*	Methanol pretreatment (Soxhlet extraction) followed by bleaching, alkaline pretreatment, and neutralization	Acid hydrolysis (H_2_SO_4_)	NCC CrI (%): 83; TS (°C): 225 °C	[85]
Industrial kelp (*Laminaria japonica*) waste	Two stages of bleaching pretreatment (Chlorine dioxide followed with hydrogen peroxide)	Acid hydrolysis (H_2_SO_4_)	NCC L: 100–500 nm; D = 20–50 nm; L/W: 5–20; Yield: 52.3%; TS (°C): 240 °C	[86]
Dealginate kelp residue From Giant Kelp (Calrose variety)	Na_2_CO_3_ (2% wt) treatment, residual sodium alginate extraction by NaOH (2% wt); Ultrasonic irradiation; NaClO_2_ (0.7% wt) buffer solution bleaching treatment and delignification	Acid hydrolysis (H_2_SO_4_)	NCC L: 100–500 nm; D = 20–50 nm; L/W: 30–70; CrI (%): 74.5; TS (°C): 120–180 °C; *l* = 120–480 nm	[87]
*Chaetomorpha antennina*	Bleaching method	Acid hydrolysis (HCl) followed with Ultrasonic irradiation	CNF E = 0.9 Gpa; CrI (%): 85.02; Y = 34.09%; TS (°C) = 200–370 °C	[88]
*Gelidium sesquipedale*	Soxhlet Extraction (Ethanol: Toluene) Bleaching treatment, delignification (5% KOH solution)	Acid hydrolysis (H_2_SO_4_) followed with neutralization (NaOH)	NCC L: 467–1650 nm; D = 18–29 nm; L/W: ~40; CrI (%): ~70%;	[89]
*Gelidium elegansred*	Alkali and bleaching pretreatment	Acid hydrolysis (H_2_SO4)	NCC L: 547.3 nm; D = 21.8 nm; L/W: 25; CrI (%): 73%; TS (°C): 334 °C	[90]
WASTE BASED AGRICULTURAL RESIDUE				
Waste sugarcane bagasse	Acidification and alkaline pretreatment	Acid hydrolysis (H_2_SO4)	NCC L: 170 nm; D = 35 nm; h = 70–90 nm; CrI (%): 93%; TS (°C): 249–345 °C	[91]
Jute dried stalks	Alkali treatment followed by steam explosion; sodium chlorite bleaching	Acid hydrolysis (oxalic acid) followed by steam explosion.	CNF L: few micrometers D = 50 nm; CrI (%): 82.2%; E: 138 Gpa; TS (°C): 250–400 °C	[92]
Coconut husk	Ultrasonic-aided solvent submersion. Delignification and Bleaching Pretreatment, followed by TEMPO-mediated Oxidation (TEMPO/NaClO/NaClO_2_; pH = 4.8)	Ultrasonication	CNF L: 150–350; D = 2–10 nm; CrI (%): 56.3%; TS (°C): 190–380 °C	[93]
Citrus waste	Alkaline and Bleaching Pretreatment	Enzymatic hydrolysis and ultrasonication	CNF L: 458 nm; W: 10.3 nm; D_avg_ = 10 nm; L/W: 47; CrI (%): 55%; TS (°C): 190–380 °C	[94]
Raw rice husk	Size Reduction, Soxhlet extraction (toluene and ethanol); Acidification (NaClO_2_ and CH_3_COOH); and delignification (5% KOH)	High pressure homogenization and high-intensity ultrasonication processes (500 W,40 min).	CNF L: 1800 nm; W: 10 nm; CrI (%): 77.5%; L/D > 180; TS (°C): 323 °C	[95]
Corn cobs	-	One pot synthesis via mechanochemical esterification	CNF σ = 110–125 MPa; E = 5.5 Gpa; D: 1.5–2.8 nm	[96]
Kenaf bast fiber	Delignification and three stage of bleaching pre-treatments	Mechanical grinder	CNF D: 1.2–34 nm; CrI (%): 82.52%; Y (%) 60.25; TS (°C): 200–400	[97]
Passion Fruit Peels	Alkaline and bleaching pretreatment	Acid hydrolysis (H_2_SO_4_) followed with ultrasonication	NCC L: 103–173.5 nm; CrI (%): 77.96%; TS (°C): 303.4; Y (%): 58.1	[29]
WASTE BASED INDUSTRIAL BY PRODUCT				
Olive industry solid waste	Pretreatment including pulping and bleaching	Acid hydrolysis (H_2_SO_4_)	NCC	[98]
Lime residues	Autoclaving pretreatment	High shear and high-pressure homogenization	CNF D: 5–28 nm; CrI (%): 44–46	[99]
Recycled Tetra Pak Food Packaging Wastes	Delignification and bleaching pretreatment	Acid hydrolysis (H_2_SO_4_) followed with ultrasonication	NCC L: 127–258 nm; D: 11.4–14 nm; L/D: 10; CrI (%): 94.8%; TS (°C): 204	[100]
Waste paper	Deinking method and alkaline pretreatment	Acid hydrolysis (H_2_SO_4_) followed with ultrasonication	NCC L: 271 nm	[101]
Discarded cigarette filters	Ethanol extraction, alkaline pretreatment, and bleaching pretreatment,	Acid hydrolysis (H_2_SO_4)_ followed with ultrasonication	NCC L: 143 nm; W: 8 nm; CrI (%): 96.77%; Y (%): 29.4	[102]
Recycled Paper Mill Sludge	Ozonation pretreatment	Acid hydrolysis (Maleic acid)	NCC L: 2431 nm; W: 165 nm; L/D: 16.7 CrI (%): 77%; Y (%): 0.8	[103]
Citrus Pulp of Floater (CPF)	Alkaline and bleaching pretreatment with autoclave	Enzymatic hydrolysis	n.d CrI (%):60	[104]
Sweet lime pulp waste	Blending and acid hydrolysis (H_2_SO_4_)	*Komagataeibacter europaeus SGP37* incubated in static intermittent fed-batch cultivation	BNC Y(g/L): CrI (%):89.6; TS (°C): 348	[105]

Abbreviation: D: Diameter; L: Length; W: Width; TS: Thermal Stability; Y: Yield; L/D: Aspect Ratio; CrI: Crystallinity Index; l: Lateral size*;* σ: Tensile strength; E: Young Modulus.

**Table 3:** Recent study of bacteria cellulose production.

Bacteria Cultivation	Source of Carbon and Its Concentration	Culture Medium	Fermentation Conditions	Yield (g/L)	References
*Komagataeib acter xylinus K2G30 (UMCC 2756)*	Glucose	GY Broth	Static; 28 °C; 9 days	6.17 ± 0.02	[118]
	Mannitol			8.77 ± 0.04	
	Xylitol			1.36 ± 0.05	
*Komagataeibacter rhaeticus PG 2*	Glycerol	Hestrin–Schramm (HS) liquid media	Static; 28 °C; 15 days	~6.9	[119]
	Glucose			~4.05	
	Sorbitol and Mannitol			~1.65–3.41	
*Komagataeibacter xylinus B12068*	Glucose	Hestrin–Schramm (HS) liquid media	Static; 30 °C; 7 days	~2.2	[120]
	Sucrose			~1.6	
	Galactose			~1.4	
	Maltose and Mannitol			~0.1–0.2	
*Komagataeibacter medellinensis*	Glucose	Standard Hestrin–Schramm (HS) Medium	Static; 28 °C; 8 days	2.80	[121]
	Sucrose			1.68	
	Fructose			0.38	
*Gluconacetobacter xylinus (PTCC 1734)*	Date syrup	Yamanaka	150 rpm; 28 °C; 7 days,	~1.15	[122]
	glucose			~0.85	
	mannitol,			~1.4	
	sucrose			~1.45	
	food-grade sucrose			~0.7	
	Date syrup	Hestrin–Schramm		~0.65	
	glucose			~0.7	
	mannitol,			~1.05	
	sucrose			~1.5	
	food-grade sucrose			~1.1	
	Date syrup	Zhou		~0.9	
	glucose			~1	
	mannitol,			~1.85	
	sucrose			~1.65	
	food-grade sucrose			~1.15	

**Table 4:** The influence of chemical functionalization on morphological nanocellulose.

Methods	Reagents	Aided Reagents	Operation Parameter	Sources of Cellulose	Mechanical Technique	Yield (%)	Morphology (nm)	CI%)	Zeta Potential (mV)	Surface Charge Density (mmol/g)	Ref.
Mineral Acids	H_2_SO_4_	-	52% H_2_SO_4_ 50 °C; 60 min	Passion Fruit Peels	Ultrasonication	58.1	NCC L: 103–173.5	77.96	−25	-	[29]
		-	63% H_2_SO_4_ 50 °C; 90 min	Microcrystalline Cellulose	Ultrasonication	30%	NCC L:250; W: 16	-	−46.1	-	[134]
		-	-	Filter Paper	-	-	NCC W: 22	85	-	-SO_3_H (0.0985)	[135]
	H_2_SO_4_/HCl	-	H_2_SO_4_:HCl:H_2_O (3:1:6); Ultrasonic 50 hZ; 10 h	Microcrystalline Cellulose	Ultrasonication	-	S-CNC (D:10–180 nm)	-	-	-	[136]
	ClSO_3_H (Post-sulfonation)	-	ClSO_3_H in 50 mL DMF; RT; 2 h	Sulfated NCC	Ultrasonication	79.31	NCC L:152; W: 22.7; h: 5.0	88%	−66.1	-SO_3_H 0.409	[137]
	H_3_PO_4_	-	73.9% H_3_PO_4_; 100 °C; 90 min	Filter Paper	Blending (15 min)	76–80	NCC L:316; W: 31;	81	-	-PO_3_ (0.0108)	[135]
		-	10.7 M H_3_PO_4_; 100 °C; 30 min	Cellulose Biotethanol Residue	Homogenizer (10 times)		NCC	83	−27	-PO_3_ (0.4352)	[138]
		-	10.7 M H_3_PO_4_; 100 °C; 30 min				CNF L: 2500 nm	81	−23	-PO_3_ (0.018)	
	H_3_PO_4_ in molten Urea	-	10.7 M H_3_PO_4_; 150 °C; 30 min				NCC L: 610 nm	83	−34	-PO_3_ (1.038)	
		-					CNF L: 330–480 nm	86	−24	-PO_3_ (1.173)	
	HCl	-	2.5 M HCl; 105 °C; 40 min	Filter Paper	Blending (40 min)	-	NCC W: 20	79%	-	-	[135]
Organic Acids	Acetic Acid	H_2_SO_4_	80 °C; 3 h	Bleached eucalyptus kraft pulp	-	81	NCC L: 264; W: 16	80	−33	-SO_3_H (0.015)	[139]
		HCl	105 °C; 9 h	Cotton	Blending (20 min)	30	NCC L: 269; W: 45	-	-	-	[140]
	Formic Acid	6M HCl	80 °C; 4 h	Microcrystalline Cellulose	-	-	NCC L: 236; W: 25	88	−1.7	Formate (0.4)	[141]
		0.015 M FeCl_3_	90 °C; 6 h	Bleached eucalyptus kraft pulp	-	75	NCC L:594	75	−6.53	Formate	[142]
	Lactic Acid	HCl	150 °C; 3 h	Cotton	Blending (20 min)	-	NCC L: 200; W = 20	80	-	Lactate	[143]
	Butyric Acid	0.027 M HCl	105 °C; 9 h	Cotton	Blending (20 min)	20	NCC L: 226; W = 34	-	-	Butyrate	[140]
	Maleic Acid (MA)	-	70% MA; 100 °C; 45 min	Bleached eucalyptus kraft pulp	-	12%	NCC	-	-33	-COOH (0.29)	[144]
		-	60% MA; 120 °C; 2 h	Bleached eucalyptus kraft pulp	Microfluidizer (120 mPa; 5 passes)	3%	L: 329.9; h = 15.9	-	−46.9	-COOH (0.368)	[145]
						84%	CNF h: 13.4	-	−45.2	-COOH (0.059)	
	Oxalic Acid (OA)	-	8.75% OA; 110 °C; 15 min	Filter paper	Sonication (60 min)	93.77	NCC L: 150–200; W: 5–20	-	−36	-COOH, 0.29	[146]
		-	70% OA; 100 °C; 1 h	Bleached eucalyptus kraft pulp	-	24.7	NCC	80	−42.5	-COOH	[144]
		-	30% OA; 100° C; 30 min	Celery	Sonication (18 min)	76.8	CNF h: 5.5	49	−32.9	-COOH	[147]
	Malonic Acid	-	80% wt of Malonic Acid; 140 °C; 3 h	Ramie Cellulose	Blending (5 min)	5%	NCC L: ~220; W: ~12	-	-	-COOH	[148]
		0.025 M HCl				19.8%		75	-	-COOH	
	Malic acid	-	80% wt of Malic Acid; 140 °C; 3 h			3.4%		-	-	-COOH, (1.617)	
		0.05 M HCl				20%		78	-	-COOH	
	Citric Acid	-	80% wt of Citric Acid; 140 °C; 3 h			5.1		-	-	-COOH	
		0.05 M HCl				20.5		78	-	-COOH, (1.884)	
		-	80% wt of Citric Acid; 100 °C; 4 h	Bleached Baggase Pulp	Ultrasonication	32	NCC, L: 251; W: 21	78	−122.9	-COOH, 0.65	[149]
		-				-	CNF, L: 654; W: 32	69	190.3	-COOH, 0.3	
Oxidation Treatment	TEMPO/NaCl /NaBr	-	TEMPO (0.094 mmol)-NaBr (1.57 mmol)- NaClO (1.24 M); 10 °C; 45 min	Nanocrystalline Cellulose	Ultrasonication	-	NCC, L: 100; W: 5–20	80%	-	-	[150]
		-	TEMPO (0.1 mmol mmol)-NaBr (1 mmol)- NaClO (5 mmol/g cellulose); Ambient condition; 1.5 h	HBKP	Ultrasonication	-	CNF	85%	-	-COOH; -CHO (1.191)	[151]
	TEMPO/O_2_/Laccase		50 mM TEMPO, 5 U mL–1 laccase; 96 h	HBKP	Ultrasonication	-	CNF, L: > 100; W: 4–8	-	-	-COOH; -CHO (0.837)	
	Sequential Periodate-Chlorite Oxidation	1 M Acetic Acid (2)	(1). 46 mmol NaIO_4_; 50 °C;4.5 h followed by (2). 12 g NaClO_2_l 50 °C; 40 h	Hardwood Pulp	Homogenizer (5 passes; 80 MPa)	-	CNF, L: 95.8; W: 2.72	-	−128	-COOH (2.0)	[152]
	APS Oxidation	-	1 M APS; 75 °C; 16 h	Cotton Linters	-	34.4	CNF, L: 95.8; W: 2.72	63.8	-	-COOH (0.16); -SO_3_ (0.98)	[153]
